# Effect of Steel Slag and Air-Cooled Blast Furnace Slag Aggregates on the Performance of Warm-Mix Asphalt Concrete Produced with Foamed Bitumen

**DOI:** 10.3390/ma19173789

**Published:** 2026-09-06

**Authors:** Justyna Stępień, Krzysztof Maciejewski, Piotr Ramiączek, Anna Chomicz-Kowalska

**Affiliations:** Department of Transportation Engineering, Faculty of Civil Engineering and Architecture, Kielce University of Technology, al. Tysiąclecia Państwa Polskiego 7, 25-314 Kielce, Poland; kmaciejewski@tu.kielce.pl (K.M.); piotrr@tu.kielce.pl (P.R.); akowalska@tu.kielce.pl (A.C.-K.)

**Keywords:** warm mix asphalt, foamed bitumen, asphalt concrete, steel slag, air-cooled blast furnace slag, water and freeze-thaw resistance, dynamic modulus, rutting resistance

## Abstract

**Highlights:**

- Foamed-bitumen warm-mix asphalt concrete for use in pavement binder courses was evaluated with 20% and 40% replacement of virgin aggregate by steel slag or air-cooled blast furnace slag.
- Slag type and replacement level affected the investigated mixture properties differently.
- Slag-containing mixtures showed a lower relative tensile strength loss after water and freeze–thaw conditioning than the reference mixtures.
- The mixture containing 20% steel slag as a replacement for virgin aggregate met all adopted technical requirements.
- Air-cooled blast furnace slag reduced dynamic modulus and rutting resistance, indicating the need for further mixture optimization.

**Abstract:**

Reducing asphalt mixture production temperatures and partially replacing virgin aggregates with industrially derived materials are potential pathways toward more sustainable pavement technologies. This study evaluated the effects of steel slag (SS) aggregate and air-cooled blast furnace slag (ACBFS) aggregate on the properties of asphalt concrete for pavement binder courses produced as warm-mix asphalt (WMA) using water-foamed bitumen. The reference hot-mix asphalt (HMA) and WMA mixtures were compared with WMA variants in which 20% or 40% of the virgin aggregate was replaced by SS or ACBFS. The slag aggregates were characterized by physical and mechanical properties, surface morphology, and local elemental composition. Mixture performance was evaluated based on air voids content, indirect tensile strength, water and freeze–thaw resistance, dynamic modulus, and rutting resistance, followed by statistical analysis. Slag type and replacement level affected the properties differently. Increasing slag content increased air voids content, whereas slag-containing mixtures showed a lower relative loss of tensile strength after conditioning than the reference mixtures. Compared with SS, ACBFS resulted in lower dynamic modulus and poorer rutting resistance. The mixture containing 20% SS satisfied all adopted technical requirements. The results support the use of SS at this replacement level, whereas ACBFS mixtures require further optimization.

## 1. Introduction

The development of materials for asphalt pavements is increasingly focused on reducing energy consumption and the use of virgin raw materials while maintaining the required performance and durability. One approach is warm-mix asphalt (WMA) technology, which enables lower mixture production and compaction temperatures than hot-mix asphalt (HMA). Another is the partial replacement of virgin aggregates with industrially derived materials. The current state of knowledge indicates that WMA can reduce energy consumption and emissions associated with mixture production, whereas the use of appropriately characterized industrial by-products can reduce the demand for virgin raw materials and improve resource efficiency [1,2,3].

Combining these two approaches may contribute to the development of more sustainable and resource-efficient pavement materials. For slag aggregates, however, the potential benefits of replacing virgin aggregate cannot be evaluated solely in terms of the quantity of industrially derived material used. Density, water absorption, porosity, mechanical resistance, and volume stability can affect mixture composition, compactability, and performance [4,5]. Integrated assessments of technologies using foamed bitumen and reclaimed materials further indicate that resource efficiency should be considered together with technical suitability and the environmental and economic consequences of the solution [6]. Increasing the proportion of industrially derived material should therefore not be treated as an objective in itself when it compromises properties relevant to pavement durability.

### 1.1. Warm-Mix Asphalt Technology with Foamed Bitumen

WMA technologies encompass various approaches for reducing mixture production and compaction temperatures, including organic and chemical additives, water-releasing materials, and water-foamed bitumen. Cheraghian et al. [1] showed that the effect of WMA depends not only on the selected technology but also on the type of bitumen and aggregate, the mixture composition, and the production and compaction conditions. Findings obtained for one WMA technology therefore cannot be directly extrapolated to another.

In foamed-bitumen technology, a small amount of water is injected into hot bitumen. Its rapid vaporization causes a temporary increase in bitumen volume and changes its processing characteristics, facilitating binder distribution and mixture compaction at reduced temperatures. For 35/50 and 50/70 paving-grade bitumens, combining water foaming with a liquid WMA additive has been shown to further reduce binder viscosity within the processing temperature range [7]. This effect was primarily associated with the production and compaction stages, with no clear evidence of permanent chemical modification of the bitumen.

Benefits observed during binder preparation and mixture production do not necessarily determine subsequent field behavior. Gao et al. [8] showed that the long-term performance of WMA can be comparable to that of HMA when climatic, structural, and service conditions are considered. Li et al. [9] reported a lower degree of aging in bitumen recovered from field-aged foamed-bitumen WMA layers, together with differences in rheological properties relative to bitumen recovered from HMA. Bairgi et al. [10] further demonstrated the importance of aging and post-construction densification for the rutting and moisture resistance of such mixtures. Reduced production temperature therefore does not eliminate the need for direct evaluation of the performance of a specific mixture.

### 1.2. Steel Slag and Air-Cooled Blast Furnace Slag Aggregates in Asphalt Concrete

Steel slag (SS) aggregate and air-cooled blast furnace slag (ACBFS) aggregate are generated at different stages of metallurgical processes and exhibit distinct properties. SS typically has high density, high mechanical resistance, angular particles, and a pronounced surface texture [4]. These characteristics can promote mechanical interlock and the formation of a stable aggregate skeleton. Previous experimental studies have also demonstrated the potential of SS as aggregate and filler in asphalt mixtures, including favorable resistance to permanent deformation and moisture-induced damage [11]. At the same time, volume stability associated with the possible presence of reactive constituents remains an important limitation on its use [5].

Using X-ray computed tomography and image analysis, Liu et al. [12] showed that SS can increase the number and length of contacts between particles within the aggregate skeleton, although the effect depends on slag content and gradation. Available findings therefore do not indicate a simple relationship between increasing SS content and improved mixture performance. Benefits associated with particle angularity and mechanical resistance may be partly offset by changes in volumetric structure, binder demand, and volume-stability-related concerns. Hassan et al. [13] reported improved resistance to permanent deformation together with an increase in optimum binder content, whereas Gan et al. [14] confirmed the importance of appropriate slag surface treatment. Liu et al. [15] also noted that greater binder demand and the higher mass of transported material may partly offset the potential environmental benefits associated with replacing virgin aggregates.

SS has previously been incorporated into WMA mixtures. Ameri et al. [16] evaluated the mechanical properties of WMA containing steel slag, whereas Hesami et al. [17] focused on moisture susceptibility. Masoudi et al. [18] investigated the behavior of steel-slag WMA after long-term aging. Amelian et al. [19] assessed moisture sensitivity and mechanical performance, while Ziaee and Behnia [20] examined dynamic and static mechanical behavior. These studies showed that favorable properties can be achieved, although the resulting effects depended on slag type and content, the property evaluated, and the WMA technology used. The investigated WMA technologies relied on additives rather than water-foamed bitumen. Their results therefore do not provide a direct basis for predicting the behavior of the mixtures investigated in the present study. They also do not establish a universal replacement level that simultaneously provides favorable volumetric characteristics, moisture resistance, stiffness, and resistance to permanent deformation.

ACBFS generally has lower density and a more porous structure than SS, which can increase water absorption and affect mixture compactability and effective binder availability. Rondón-Quintana et al. [21] demonstrated the feasibility of using blast furnace slag as a replacement for the fine aggregate fraction. In subsequent work, they compared coarse aggregate replacement on a mass and volume basis and highlighted the importance of density differences in determining the actual slag proportion within the aggregate skeleton [22].

The evidence base for ACBFS in WMA is considerably more limited than that for SS. Ruíz-Ibarra et al. [23] investigated blast furnace slag as a partial replacement for the coarse aggregate fraction at replacement levels of 12.5% and 24% in mixtures produced with a chemical WMA additive. Improved performance was reported at the lower replacement level, whereas less favorable results were obtained at 24%. Comparisons with the present study are also limited by differences in WMA technology and aggregate dosage, particularly because equal mass fractions do not necessarily correspond to equal volume fractions within the aggregate skeleton [22]. Available results therefore do not allow the effects of higher ACBFS contents in water-foamed WMA asphalt concrete to be determined unequivocally.

### 1.3. Research Gap, Objective, and Novelty

Despite advances in WMA and the use of slag aggregates, knowledge remains limited regarding the combination of these approaches in WMA produced with water-foamed bitumen. SS has already been used in WMA, but previous studies employed other technological solutions [16,17,18,19,20]. Even less information is available for ACBFS in WMA, and the available study used a chemical additive to reduce production temperature [23].

No studies were identified in the available literature that directly compared SS and ACBFS in the same WMA asphalt concrete produced with water-foamed bitumen at multiple virgin aggregate replacement levels for both slag types. This gap is also relevant to resource-efficient mixture design because increasing the proportion of industrially derived material may reduce the demand for virgin aggregate but does not necessarily result in a more favorable solution if material performance deteriorates simultaneously.

The objective of this study was to evaluate the effects of slag aggregate type and virgin aggregate replacement level on the properties of foamed-bitumen WMA asphalt concrete intended for pavement binder courses subjected to traffic loading in the range 0.50 < ESAL_100 kN_ ≤ 7.30 million equivalent 100 kN standard axles per design lane [24]. SS and ACBFS were compared at replacement levels of 20% and 40% by mass of the aggregate blend. Reference HMA and WMA mixtures were also included, enabling the combined effect of the adopted WMA technology to be assessed separately from the effects of slag aggregate type and content.

It was hypothesized that the differences in the physical, mechanical, and morphological properties of SS and ACBFS would result in different WMA mixture responses and that the investigated properties would also depend on the virgin aggregate replacement level. This study also examined whether the proportion of industrially derived aggregate could be increased without loss of compliance with the adopted technical requirements.

The novelty of this study lies in the parallel comparison of SS and ACBFS at two replacement levels in the same foamed-bitumen WMA asphalt concrete. This experimental framework makes it possible to assess whether reduced production temperature can be combined with partial replacement of virgin aggregate by industrially derived materials while maintaining the required mixture performance. A complete assessment of environmental benefits, however, requires a separate life-cycle analysis and is beyond the scope of the present study.

## 2. Materials and Methods

### 2.1. Experimental Program

The experimental program was developed to determine the effects of slag aggregate type and content on the properties of asphalt concrete intended for pavement binder courses and produced as WMA using foamed bitumen. Two slag aggregate types were investigated: steel slag aggregate, designated SS, and air-cooled blast furnace slag aggregate, designated ACBFS. An HMA mixture was also included as a reference. Six mixtures were designed and tested:

- HMA-REF, a reference hot-mix asphalt mixture;
- WMA-REF, a reference WMA mixture produced with foamed bitumen;
- WMA-SS20 and WMA-SS40, WMA mixtures containing 20% and 40% SS aggregate, respectively;
- WMA-ACBFS20 and WMA-ACBFS40, WMA mixtures containing 20% and 40% ACBFS aggregate, respectively.

The SS and ACBFS contents were defined relative to the total mass of the aggregate blend. The proportions of individual aggregate constituents were adjusted to maintain a gradation as close as possible to that of the reference mixture and to limit the influence of gradation changes on the evaluated properties.

HMA-REF and WMA-REF had the same aggregate blend and binder content. They differed in production and compaction temperatures, binder preparation method, and adhesion promoter content. Their comparison therefore enabled evaluation of the combined effect of the adopted WMA technology, including reduced production and compaction temperatures, water foaming of the bitumen, and increased adhesion promoter content.

**Figure 1:**
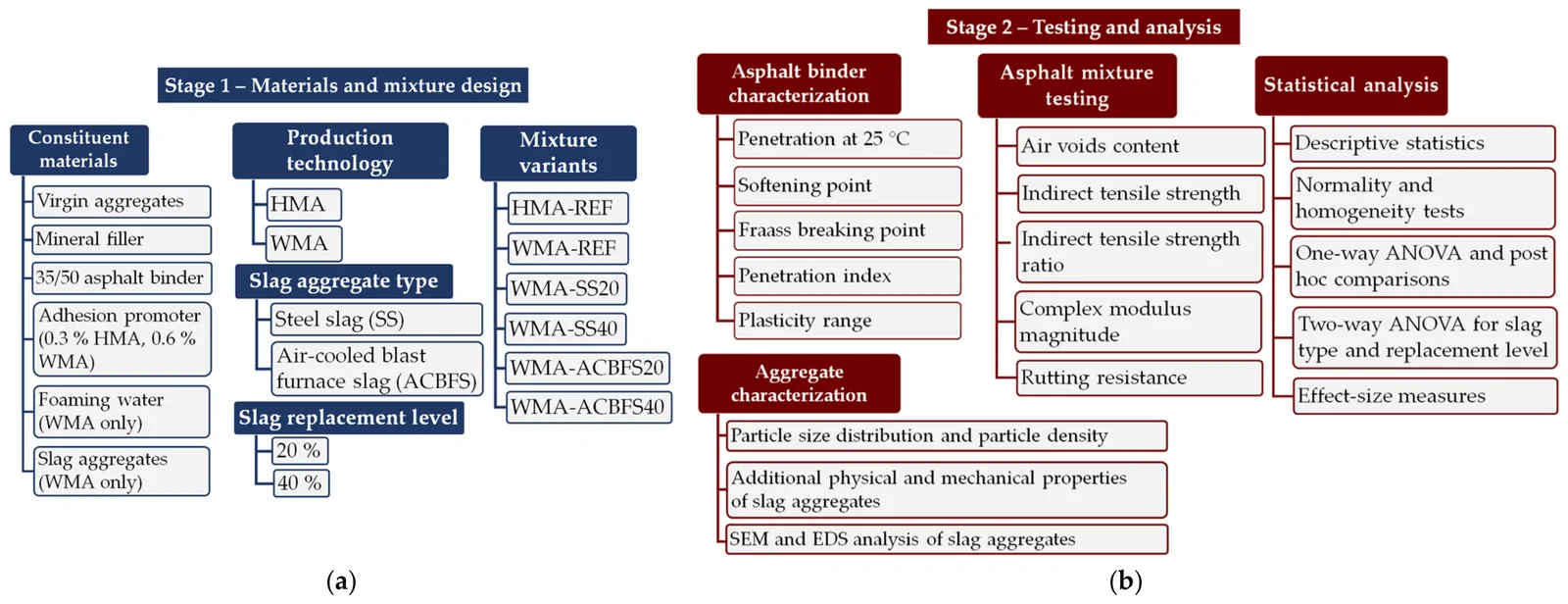
Experimental program of this study: (**a**) materials and mixture design; (**b**) laboratory testing and statistical analysis.

The overall experimental program is shown in Figure 1.

The program included characterization of the constituent materials, including the basic properties of the bitumen and its foaming characteristics, as well as the properties of the slag aggregates together with SEM and EDS analyses of their surface morphology and local elemental composition. Mixture compositions were then designed, production and compaction conditions were established, and specimens were prepared for testing. Mixture performance was evaluated based on air voids content, indirect tensile strength and resistance to water and freeze–thaw conditioning, dynamic modulus, and rutting resistance. The results were subjected to statistical analysis, including descriptive statistics and one-way and two-way analyses.

### 2.2. Materials

#### 2.2.1. Virgin Aggregates and Mineral Filler

For asphalt mixture design, the mineral constituents were selected in accordance with EN 13043 [25] and the applicable technical requirements [26]. Mixture design was performed in accordance with EN 13108-1 [27] and the technical requirements [28]. The aggregate blend consisted of limestone mineral filler, 0/2 and 0/4 mm limestone aggregates, 2/5 and 5/8 mm quartzite aggregates, and 8/16 mm limestone aggregate. The limestone mineral filler and limestone aggregates were supplied by Trzuskawica S.A. (Nowiny, Poland), whereas the quartzite aggregates were obtained from Eurovia Kruszywa S.A., Wiśniówka Quarry (Zagnańsk, Poland). The mineralogical type, size fraction, and particle density of the materials are summarized in Table 1.

The particle size distribution of the mineral filler was determined by air-jet sieving in accordance with EN 933-10 [29], whereas the remaining aggregates were tested by sieving in accordance with EN 933-1 [30]. Aggregate particle densities were determined in accordance with EN 1097-6 [31], and mineral filler density in accordance with EN 1097-7 [32].

**Table 1:** Virgin aggregates and mineral filler used in the investigated mixtures.

Material	Aggregate Size (mm)	Mineralogical Type	Particle Density (Mg/m^3^)	Test Method
Mineral filler	<0.063	Limestone	2.71	EN 1097-7 [32]
Fine aggregate	0/2	Limestone	2.72	EN 1097-6 [31]
All-in aggregate	0/4	Limestone	2.69	EN 1097-6 [31]
Coarse aggregate	2/5, 5/8	Quartzite	2.66	EN 1097-6 [31]
	8/16	Limestone	2.70	EN 1097-6 [31]

#### 2.2.2. Slag Aggregates

**Figure 2:**
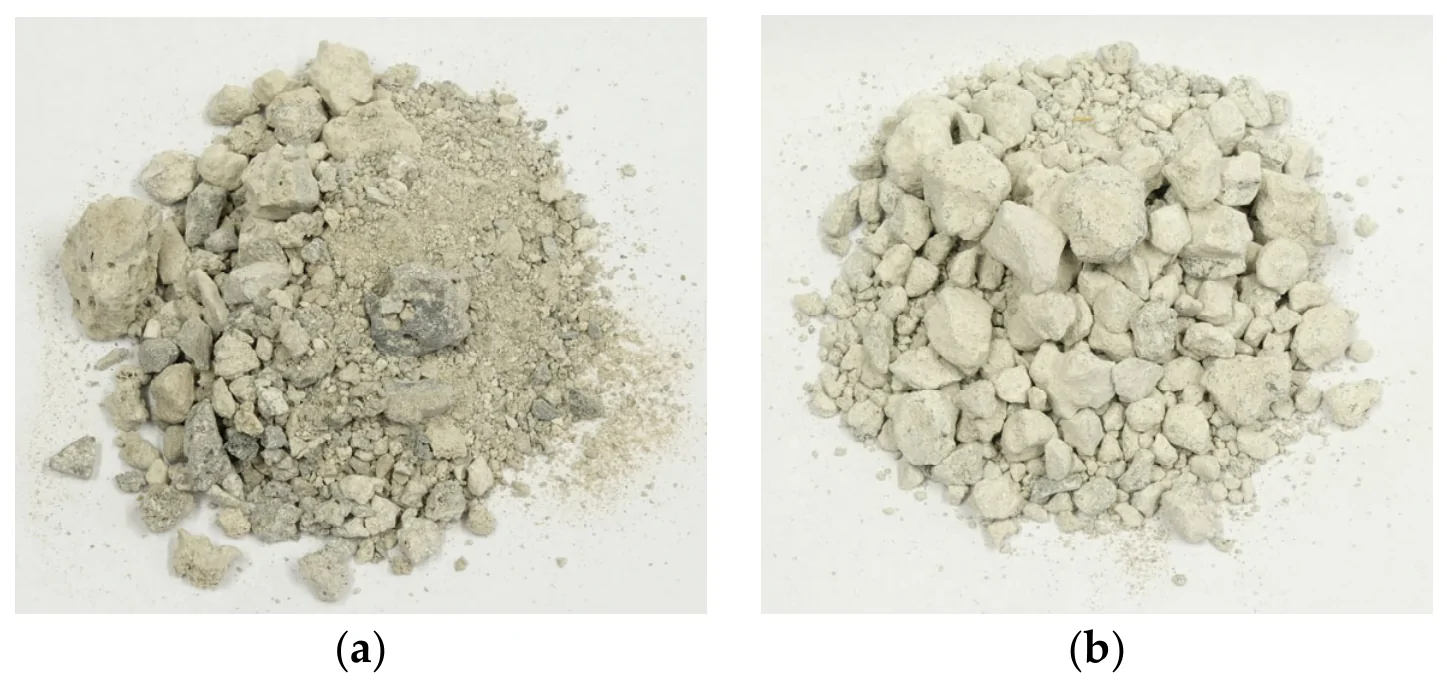
Macroscopic appearance of the investigated slag aggregates: (**a**) ACBFS; (**b**) SS.

This study used 0/16 mm manufactured aggregates derived from SS and ACBFS. The SS aggregate originated from electric arc furnace (EAF) steelmaking and was obtained from Ostrowiec Świętokrzyski, Poland (HARSCO Metals Polska Sp. z o.o.). According to the information provided by the producer, steel slag aggregate from current production is aged on a stabilization stockpile for at least six months before use. The SS investigated in this study had also been stockpiled for several months before testing. The ACBFS aggregate originated from air-cooled blast-furnace slag generated during pig-iron production and was obtained from the Pleszów slag dump in Kraków, Poland (Ekoprod Sp. z o.o.), where the material had been stockpiled prior to its use in this study. These materials originated from waste streams of the iron and steel industry, classified in the European List of Waste under group 10 02 (wastes from the iron and steel industry) [33]. The macroscopic appearance of the investigated slag aggregates was documented by digital photography and is shown in Figure 2.

The particle size distributions of the slag aggregates were determined in accordance with EN 933-1 [30]. The geometrical, physical, and mechanical characterization included fines content, fines quality, resistance to fragmentation and wear, particle density, water absorption, freeze–thaw resistance, and properties related to volume stability. The test methods and results are summarized in Table 2.

The suitability of the slag aggregates was assessed in accordance with EN 13043 [25] and the technical requirements [26]. The slag-specific disintegration and volume-stability requirements were satisfied. ACBFS showed resistance to dicalcium silicate and iron disintegration, whereas SS met the volume stability requirement. The aggregates differed, however, in resistance to fragmentation. SS had a Los Angeles coefficient of 23, satisfying the LA30 category adopted for binder course applications within the analyzed traffic loading range. ACBFS had LA = 35 and therefore did not satisfy the LA30 requirement adopted for the target traffic loading. Consequently, the investigated ACBFS aggregate was not eligible for the intended binder-course application, irrespective of the mixture-level results. It was retained in the experimental program to assess its mixture-level behavior and its potential use under lower traffic loading or after optimization of the aggregate properties. SS also had higher particle density and lower water absorption than ACBFS.

**Figure 3:**
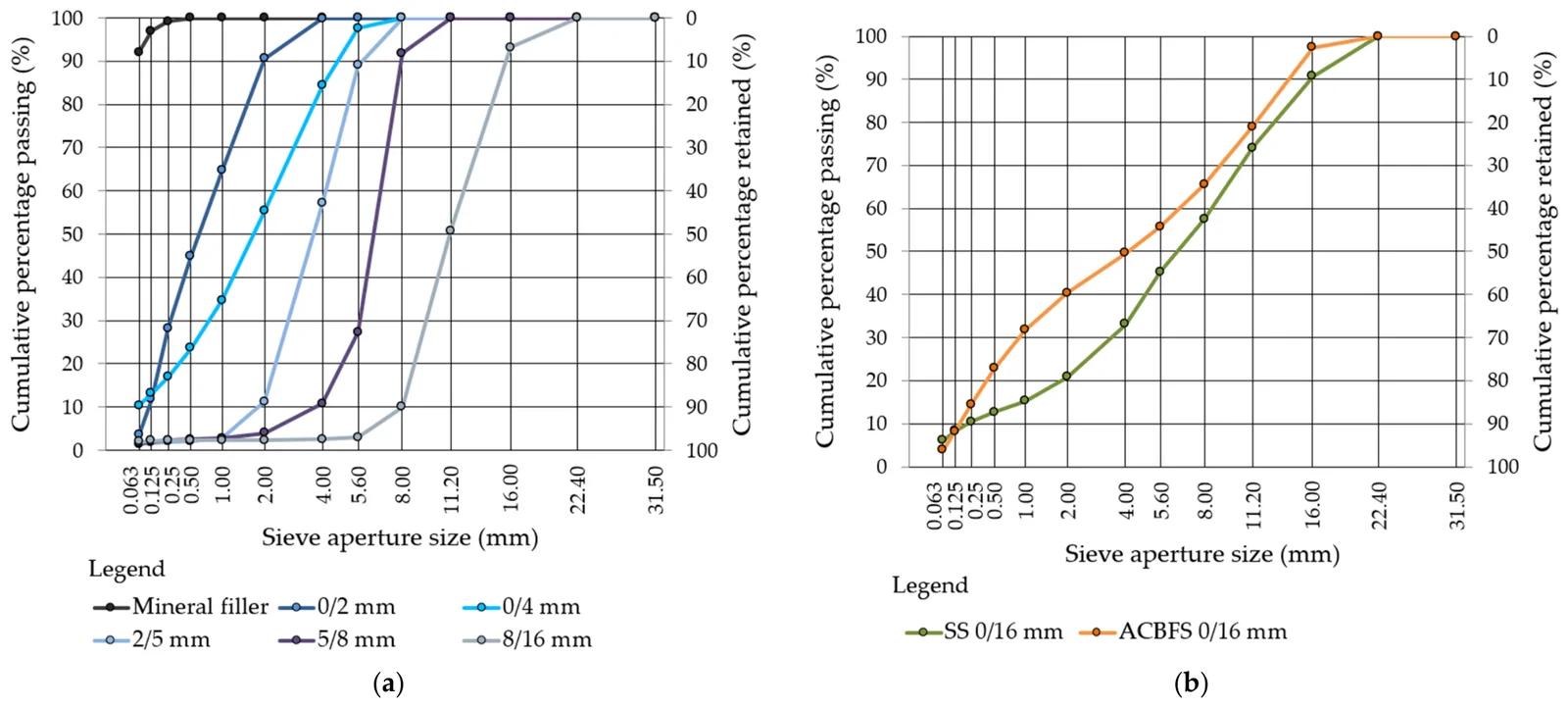
Particle size distributions of the aggregate constituents used in the investigated mixtures: (**a**) virgin aggregates and mineral filler; (**b**) SS and ACBFS slag aggregates.

The particle size distributions of the virgin aggregates, mineral filler, and slag aggregates are shown in Figure 3.

#### 2.2.3. Asphalt Binder, Adhesion Promoter, and Foamed Bitumen Characteristics

A 35/50 paving-grade bitumen (ORLEN Asfalt Sp. z o.o., Gdańsk, Poland) complying with EN 12591 [41] was used in all mixtures. A liquid adhesion promoter Wetfix BE (Nouryon, Amsterdam, The Netherlands), with adhesion-promoting and WMA-supporting functions was incorporated into the bitumen. The additive was a brown, viscous liquid with a density of 0.98 Mg/m^3^ at 20 °C, an amine value of 159–185 mg HCl/g, and a flash point above 218 °C. Its intended functions were to improve bitumen foamability, mixture workability and compactability, and binder–aggregate affinity. The adhesion promoter content was 0.3% by mass of bitumen in HMA-REF and 0.6% in all WMA mixtures. The basic characteristics of the base bitumen and the bitumen containing the adhesion promoter were determined from penetration at 25 °C in accordance with EN 1426 [42], Ring-and-Ball softening point in accordance with EN 1427 [43], and Fraass breaking point in accordance with EN 12593 [44]. The penetration index and plasticity range were also calculated. The results are summarized in Table 3.

All tested binders met the relevant EN 12591 [41] requirements for 35/50 paving-grade bitumen. Addition of the adhesion promoter at 0.3% and 0.6% by mass of bitumen increased penetration by 4.8 and 6.4 × 0.1 mm, respectively, while slightly reducing the softening point by 0.6 and 0.4 °C. The Fraass breaking point increased by 0.3 °C at 0.3% adhesion promoter and by 1.1 °C at 0.6%. The penetration index changed from −1.1 to −1.0 and −0.9, whereas the plasticity range decreased from 67.0 °C to 66.1 °C and 65.5 °C. These results indicate that the additive did not cause substantial changes in the basic properties of the bitumen.

The 35/50 bitumen containing 0.6% adhesion promoter was foamed with water using a WLB 10S laboratory foaming unit (Wirtgen GmbH, Windhagen, Germany). The bitumen temperature before foaming was maintained at 155 °C, and the foaming water temperature was approximately 20 °C. Air and water pressures were 500 and 600 kPa, respectively. Foamed-bitumen characteristics were evaluated using the expansion ratio (ER) and half-life (HL). Measurements were performed at foaming water contents (FWCs) of 1.5%, 2.5%, and 3.5% by mass of bitumen. Four replicate measurements were performed at each FWC. Based on the trends in ER and HL as functions of FWC, an FWC of 3.0% was selected for mixture production as a compromise between expansion ratio and half-life. For 35/50 bitumen containing 0.6% adhesion promoter, ER = 14.0 and HL = 20.0 s were obtained at FWC = 3.0%. For comparison, the base 35/50 bitumen at the same FWC had ER = 14.0 and HL = 24.0 s.

### 2.3. SEM and EDS Analysis of Slag Aggregates

The surface morphology and local elemental composition of the SS and ACBFS aggregates were examined using a Quanta FEG 250 scanning electron microscope (FEI Company, Hillsboro, OR, USA) equipped with an Apollo XL silicon drift detector (EDAX Inc., Mahwah, NJ, USA) for energy-dispersive X-ray spectroscopy. Separate aggregate specimens were prepared for SEM imaging and EDS analysis. Specimens used for SEM imaging were mounted on specimen holders and coated with a thin gold layer to provide electrical conductivity, whereas EDS measurements were performed on uncoated specimens.

SEM observations and EDS measurements were performed at an accelerating voltage of 20 kV. SEM images used for morphological assessment were acquired at a working distance of approximately 9.6–9.9 mm and magnifications of 500× and 5000×. EDS measurements were conducted at a take-off angle of approximately 43.7–43.9° and an acquisition time of 240 s. Two randomly selected surface areas were analyzed for each slag aggregate. The two areas were analyzed separately, and the results were reported individually, without averaging them to represent the bulk composition. Standardless quantification with ZAF correction was applied, and the detected elemental contents were normalized to 100 wt.%. The EDS results were interpreted as a local, semi-quantitative characterization of surface composition.

### 2.4. Asphalt Mixture Design and Composition

The mixtures were designed as AC 16 asphalt concrete, with a nominal maximum aggregate size of 16 mm, for pavement binder course applications in accordance with EN 13108-1 [27] and the technical requirements [28]. The adopted requirements corresponded to traffic loading in the range 0.50 < ESAL_100 kN_ ≤ 7.30 million equivalent 100 kN standard axles per design lane [24]. The gradation of each aggregate blend was selected so that the gradation curve remained within the specified envelope for the adopted asphalt concrete type according to the technical requirements [28]. The adopted technical requirements reflect service conditions characteristic of Poland, including seasonal temperature variations, subzero temperatures and freeze–thaw cycles, and elevated pavement temperatures during summer.

The same aggregate blend was used in HMA-REF and WMA-REF. In the remaining variants, SS or ACBFS accounted for 20% or 40% of the aggregate blend mass. Because SS and ACBFS have different particle densities, the corresponding volumetric composition of the aggregate blends was additionally calculated from the mass proportions and particle densities of the individual aggregate fractions. The mixture design was based on mass proportions, while the volumetric fractions are provided to facilitate comparison between mixtures containing slag aggregates of different densities. The proportions of the individual virgin aggregate fractions were adjusted separately for each variant to maintain continuous gradation and satisfy the specified grading limits.

The aggregate blend compositions, binder content, adhesion promoter content, and foaming water content are summarized in Table 4.

The binder content was selected according to the minimum binder content specified in the adopted technical requirements [28], in accordance with EN 13108-1 [27]. For the investigated asphalt concrete mixture intended for the binder course, the specified minimum binder content, $B_{min}$, is 4.6% for an aggregate blend density of 2.650 Mg/m^3^. For aggregate blends with a different density, $B_{min}$ was corrected using the following equations:

(1) $$\alpha \;=\frac{2.650}{\rho_{a}},$$

(2) $$B_{min,corr}=B_{min}\times \alpha ,$$

where $\rho_{a}$ is the particle density of the aggregate blend and $B_{\min,corr}$ is the minimum binder content corrected for the aggregate blend density. This correction resulted in binder contents of 4.5% for HMA-REF, WMA-REF, WMA-ACBFS20, and WMA-ACBFS40, 4.3% for WMA-SS20, and 4.1% for WMA-SS40.

### 2.5. Mixture Production and Specimen Compaction

Before mixture production, the mineral constituents were conditioned at the target temperature for 4 h. Processing conditions differed according to mixture type:

- HMA-REF: Aggregates and bitumen were heated to 170 °C. The mixture was produced at 160–170 °C, and specimens were compacted at 140–145 °C.
- All WMA mixtures: Before foaming, the bitumen was heated to 155 °C. The mixtures were produced at 130–135 °C, and specimens were compacted at 110–115 °C.

The adhesion promoter was introduced into the bitumen approximately 1 h before mixture preparation and blended until a homogeneous binder was obtained. For WMA mixtures, the foamed bitumen was added to the heated aggregate blend immediately after completion of the foaming process.

### 2.6. Asphalt Mixture Testing Methods

The test methods used to evaluate the investigated asphalt mixtures, together with specimen numbers, basic test conditions, measured parameters, and units, are summarized in Table 5.

Air voids content, $V_{a}$, was determined from the maximum density of the asphalt mixture and the bulk density of compacted specimens. Maximum density was determined in accordance with EN 12697-5 [45], bulk density in accordance with EN 12697-6 [46], and $V_{a}$ was calculated in accordance with EN 12697-8 [47]. Marshall specimens were compacted using an automatic impact compactor, model 76-B4432 (CONTROLS Group, Liscate, Italy).

The indirect tensile strength ratio (ITSR) was calculated as the ratio of the mean indirect tensile strength of specimens after water and freeze–thaw conditioning, ITS_w_, to the mean indirect tensile strength of dry specimens, ITS_d_, expressed as a percentage. This parameter represented the relative retention of strength after conditioning and was interpreted together with the absolute ITS_d_ and ITS_w_ values.

Dynamic modulus, |E*|, was determined using a UTM-25 universal testing machine (IPC Global, CONTROLS Group, Liscate, Italy) under uniaxial cyclic compression at 13 °C and a loading frequency of 10 Hz. Specimens were compacted using a GYROCOMP PLUS gyratory compactor (CONTROLS Group, Liscate, Italy). The testing temperature of 13 °C was selected as the equivalent temperature adopted for asphalt layers in flexible pavement structures under Polish climatic conditions [24]. This value was established based on the analysis of 30 years of air-temperature data from 22 monitoring stations in Poland and pavement structural calculations. Dynamic modulus was therefore used as a comparative measure of mixture stiffness under the adopted reference temperature and loading conditions.

Test slabs were compacted using a Dyna-Comp roller compactor, model 77-B3602 (CONTROLS Group, Liscate, Italy). Rutting resistance was evaluated using a Dyna-Track wheel-tracking apparatus, model 77-B3502 (CONTROLS Group, Liscate, Italy), based on proportional rut depth, PRD_AIR_, and wheel-tracking slope, WTS_AIR_. PRD_AIR_ represented the final rut depth normalized by the initial specimen thickness and expressed as a percentage. WTS_AIR_ characterized the rate of rut depth increase during the final portion of the test. Lower values of both parameters indicated greater resistance to permanent deformation.

### 2.7. Statistical Analysis

The data were compiled, organized, and initially screened using Microsoft Excel 2019 (Microsoft Corporation, Redmond, WA, USA). The software was also used to prepare plots presenting the test results and statistical analyses. Descriptive statistics included the mean, standard deviation, coefficient of variation, and 95% confidence interval for the mean. Normality within each group was assessed using the Shapiro–Wilk test, whereas homogeneity of variance was evaluated using the Brown–Forsythe test.

Differences among the six investigated mixtures were evaluated using one-way analysis of variance (one-way ANOVA). When the assumption of homogeneity of variance was satisfied, Tukey’s HSD test was used for multiple comparisons. When this assumption was not satisfied, Welch’s analysis of variance followed by the Games–Howell test was applied. Where justified, the parametric analysis was supplemented by the Kruskal–Wallis test to verify the consistency of the overall statistical conclusion. The effect size for one-way ANOVA was quantified using omega squared, $\omega^{2}$.

For the four WMA mixtures containing slag aggregates, a two-way analysis of variance (two-way ANOVA) was additionally performed. The factors were slag aggregate type (SS or ACBFS) and virgin aggregate replacement level (20% or 40%). For each response variable, a fixed-effects two-way ANOVA model including the main effects of slag aggregate type and replacement level and their interaction was fitted:

(3) $$Y_{ijk}=\;\mu +\alpha_{i}+\beta_{j}+{\left(\alpha \beta \right)}_{ij}+\varepsilon_{ijk},$$

where $Y_{ijk}$ is the observed value, μ is the overall mean, $\alpha_{i}$ is the effect of slag aggregate type, $\beta_{j}$ is the effect of replacement level, ${\left(\alpha \beta \right)}_{ij}$ is the interaction between the two factors, and $\varepsilon_{ijk}$ is the random error term. Effect sizes were expressed as partial eta squared ($\eta_{p}^{2}$). Because each factor comprised only two levels, significant main effects directly represented differences between the two corresponding factor levels and therefore did not require additional post hoc comparisons. Simple-effects comparisons were planned only in the event of a significant interaction.

Statistical calculations, except for the Games–Howell test, were performed using Statistica 13.3 (TIBCO Software Inc., Palo Alto, CA, USA). The Games–Howell test was conducted in R 4.6.1 (R Foundation for Statistical Computing, Vienna, Austria) using the gamesHowellTest() function from the PMCMRplus 1.9.12 package. Statistical significance was assessed at α = 0.05.

## 3. Results

### 3.1. Morphological and Elemental Characterization of Slag Aggregates

**Figure 4:**
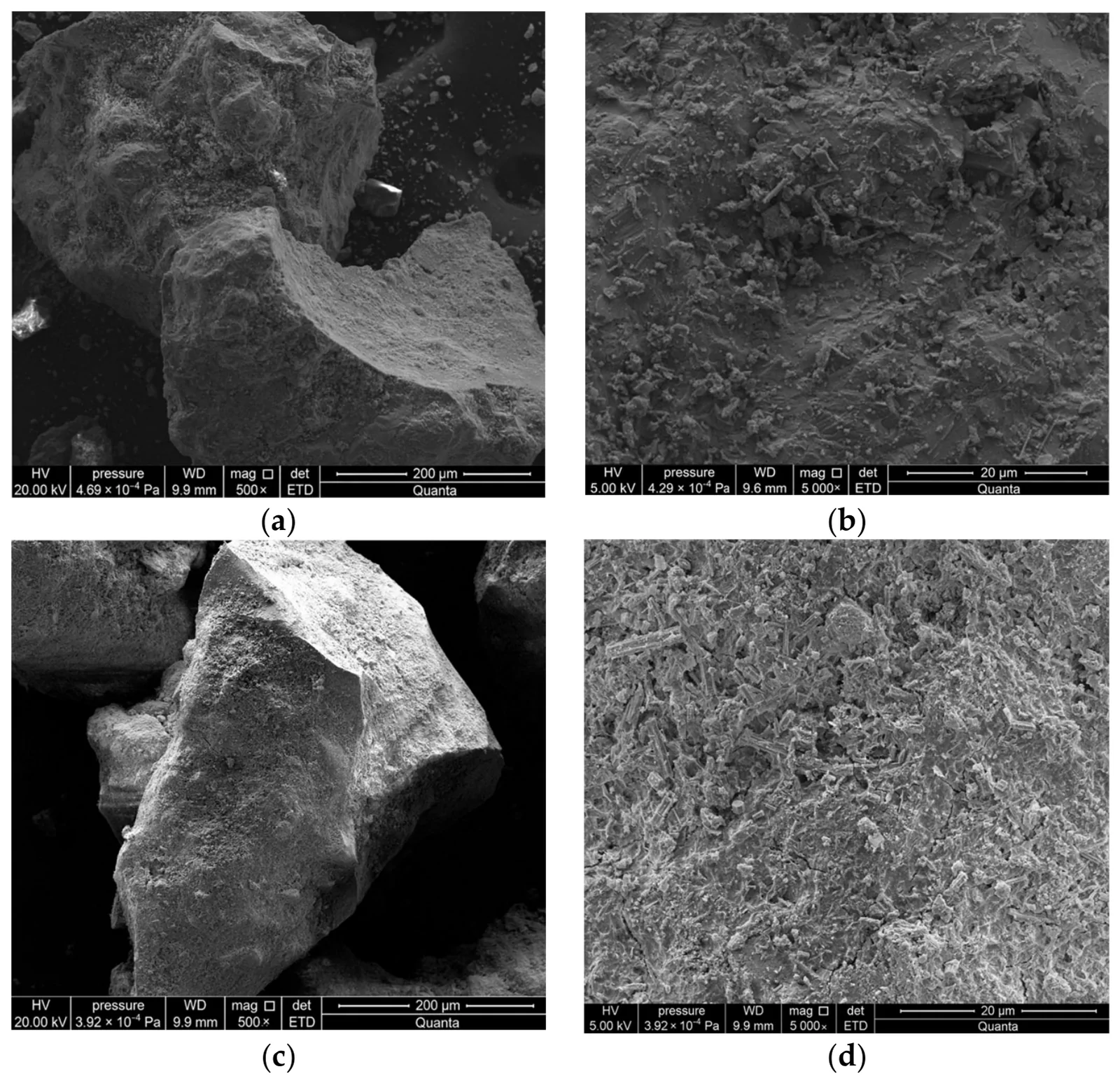
SEM micrographs of the investigated slag aggregates: (**a**) SS at 500× magnification; (**b**) SS at 5000× magnification; (**c**) ACBFS at 500× magnification; and (**d**) ACBFS at 5000× magnification.

The surface morphology of the SS and ACBFS aggregates was evaluated from SEM images acquired at magnifications of 500× and 5000×. Selected micrographs are shown in Figure 4. The lower magnification was used to assess overall particle shape and surface variability, whereas the higher magnification was used to compare surface microtexture.

Both aggregate types exhibited irregular, angular particles and well-developed surface textures. The SS surface was heterogeneous and included dense fracture areas, fine irregular fragments, and local depressions. At 5000× magnification, a complex microtexture formed by particles of varying sizes and shapes was visible.

ACBFS particles also exhibited angular geometry, but their surfaces showed greater textural heterogeneity. At higher magnification, a fine, highly developed surface structure with numerous irregular features and local voids and depressions was observed. The SEM images therefore indicate differences in the micromorphology of the two aggregates, with ACBFS exhibiting a more pronounced fine-scale texture within the analyzed field of view. The local elemental composition of the aggregate surfaces was determined by EDS. Results for two analyzed areas of each slag type are presented in Table 6. Because EDS is a local, semi-quantitative method, the results characterize selected surface areas rather than the bulk chemical composition of the entire aggregate batch.

In the analyzed SS areas, Fe, O, and Ca were the dominant elements. Fe contents ranged from 27.75 to 34.25 wt.%, Ca from 19.27 to 22.05 wt.%, and O from 22.71 to 31.14 wt.%. Si, Al, Mn, and Mg were also detected, together with smaller amounts of Na, S, Ti, and Cr. The results obtained for the two analyzed areas indicate local compositional variation in SS, particularly with respect to Fe, O, and Al. The Fe–Ca–O-rich local composition of the investigated SS is consistent with its EAF origin. Previous WMA studies cited for comparison also investigated EAF steel slag [16,18,20], although direct chemical equivalence between slags from different sources cannot be assumed because their composition is process- and source-dependent.

In the analyzed ACBFS areas, the dominant elements were O, Ca, and Si. O contents ranged from 35.49 to 39.03 wt.%, Ca from 33.84 to 35.44 wt.%, and Si from 16.14 to 16.43 wt.%. Smaller amounts of Mg, Al, S, K, and Na were detected. The results from the two analyzed ACBFS areas were similar, particularly in terms of Ca and Si contents.

**Figure 5:**
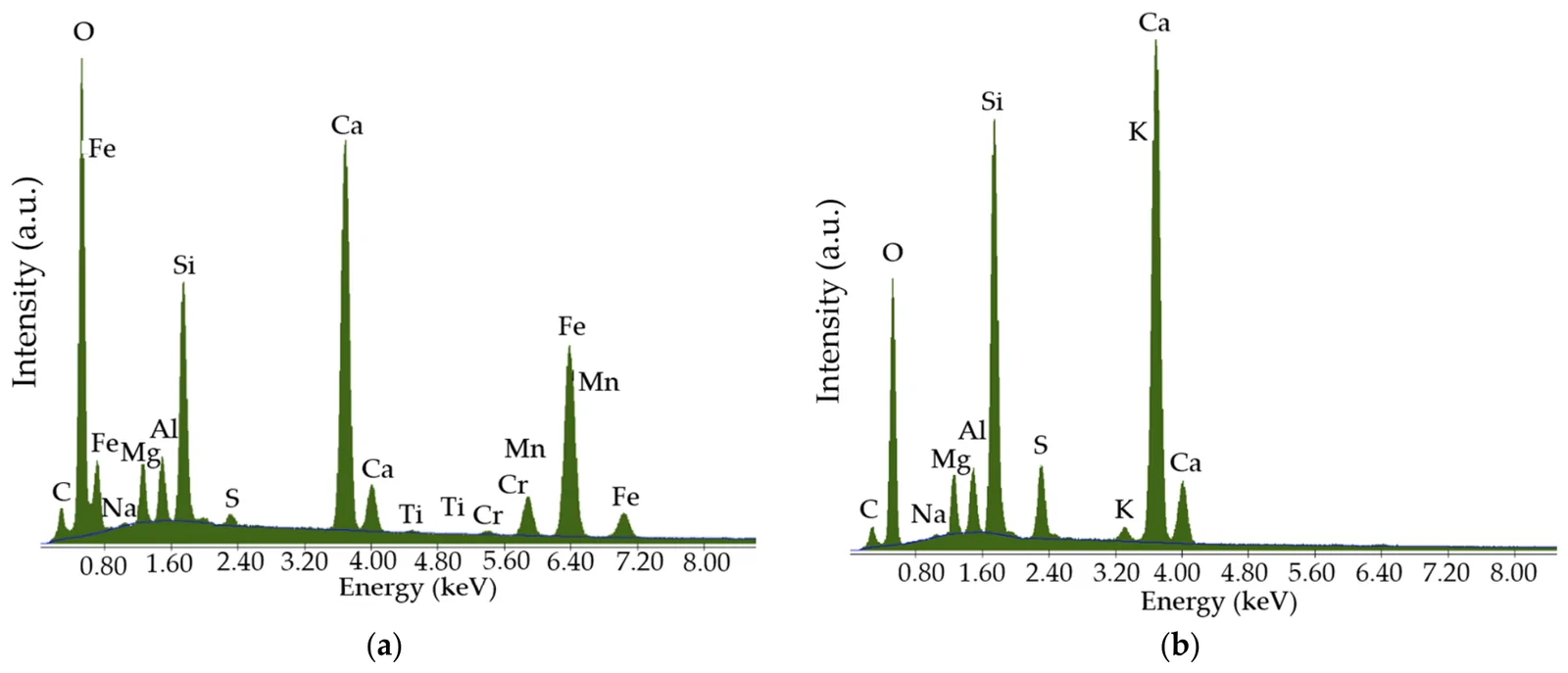
Representative energy-dispersive X-ray spectroscopy spectra obtained from selected surface areas of the investigated slag aggregates: (**a**) SS, sample 1; (**b**) ACBFS, sample 1.

Representative EDS spectra of both aggregates are shown in Figure 5. The SS spectrum exhibited pronounced Fe, Ca, O, and Si peaks, whereas the ACBFS spectrum was dominated by Ca, Si, and O peaks. The spectra reflected the differences in the local elemental composition of the investigated surfaces.

### 3.2. Air Voids Content

**Figure 6:**
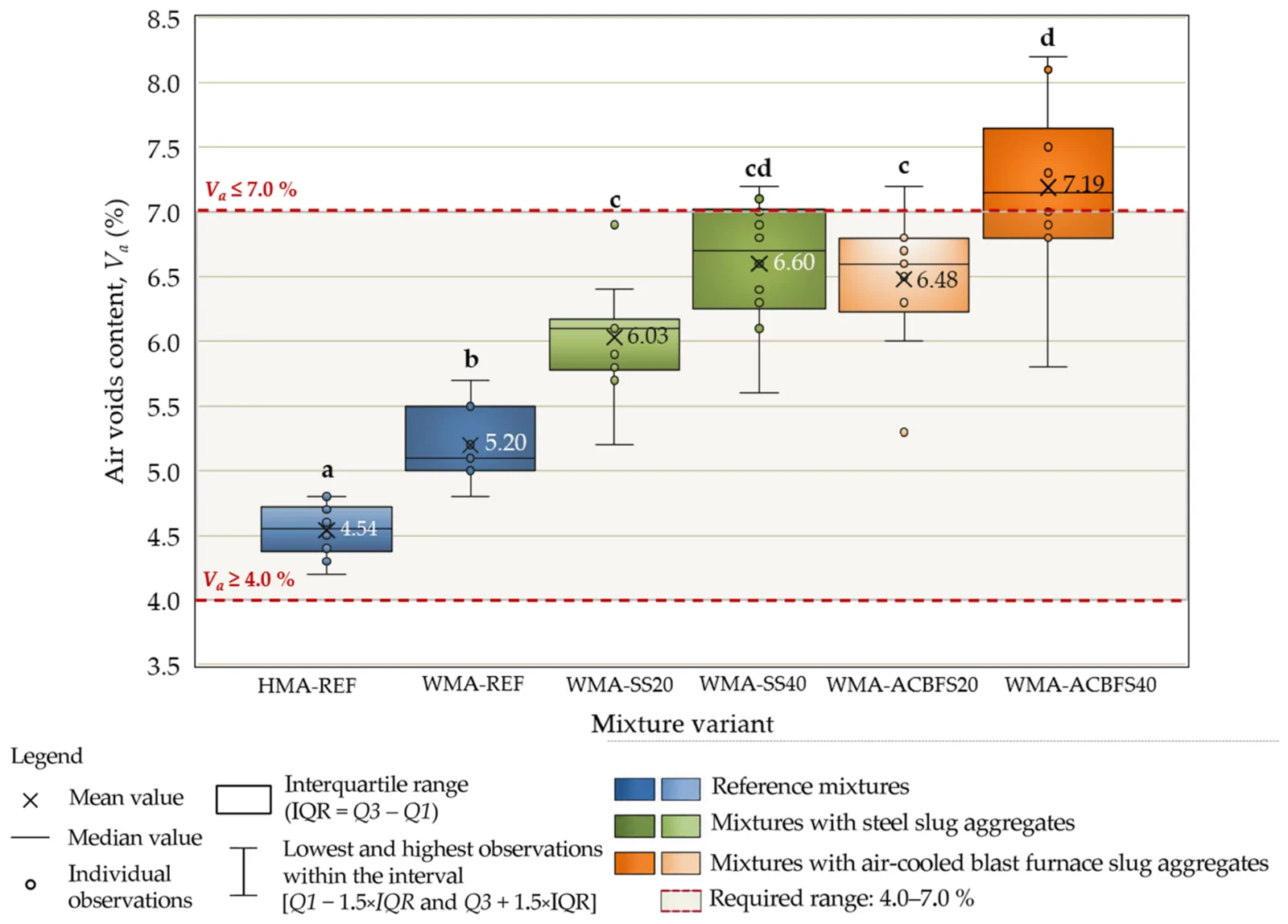
Air voids content of the investigated mixtures. Lowercase letters indicate homogeneous groups determined using Tukey’s HSD test at α=0.05 (mean values sharing at least one letter are not significantly different).

Descriptive statistics for air voids content and the results of Tukey’s HSD multiple comparisons are presented in Table 7, whereas the distributions and identified homogeneous groups are shown in Figure 6.

WMA-REF had a mean *V_a_* value 0.66 percentage points higher than HMA-REF. Incorporation of slag aggregates produced a further increase in air voids content, and this effect became more pronounced as the replacement level increased. Relative to WMA-REF, the increase in mean *V_a_* ranged from 0.83 to 1.99 percentage points. At both replacement levels, mixtures containing ACBFS had higher *V_a_* values than the corresponding mixtures containing SS.

Increasing the slag aggregate replacement level from 20% to 40% increased mean *V_a_* by 0.57 percentage points for SS and 0.71 percentage points for ACBFS. Except for WMA-ACBFS40, the mean values were within the required range of 4.0–7.0% [28]. WMA-ACBFS40 slightly exceeded the upper *V_a_* limit, with a mean value of 7.19%. The greatest result variability was observed for WMA-ACBFS40, with a coefficient of variation of 9.73%.

The Shapiro–Wilk test showed no significant departures from normality in any mixture group (p=0.208–0.717), and the Brown–Forsythe test did not indicate heterogeneity of variance (p=0.117). One-way ANOVA showed significant differences among mixture variants in *V_a_*, F(5,54)=43.04, p<0.001, $\omega^{2}=0.778$.

Tukey’s HSD test confirmed a difference between HMA-REF and WMA-REF and between WMA-REF and all slag-containing variants. At the same replacement level, differences between SS and ACBFS mixtures were not statistically significant. Increasing SS content from 20% to 40% also did not cause a significant change in *V_a_*, whereas increasing the ACBFS content resulted in a significant increase in this parameter. WMA-ACBFS40 differed significantly from WMA-SS20 and WMA-ACBFS20 but not from WMA-SS40.

### 3.3. Indirect Tensile Strength and Water and Freeze–Thaw Resistance

**Figure 7:**
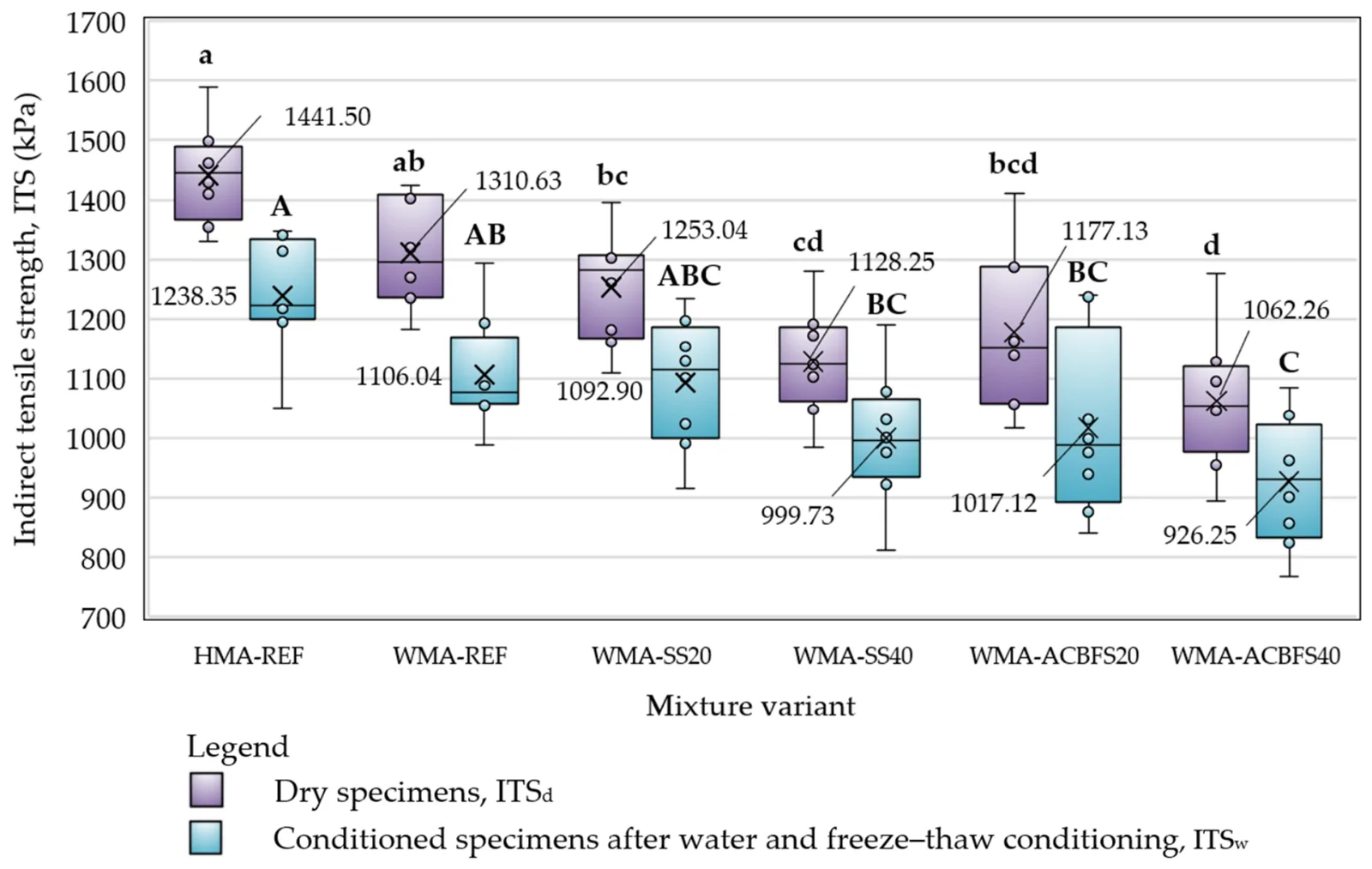
Indirect tensile strength of the investigated mixtures determined for dry specimens (ITS_d_) and conditioned specimens after water and freeze–thaw conditioning (ITS_w_). Lowercase and uppercase letters denote homogeneous groups for ITS_d_ and ITS_w_, respectively, according to Tukey’s HSD test at α=0.05. The boxplot elements are defined as in the legend to Figure 6.

**Figure 8:**
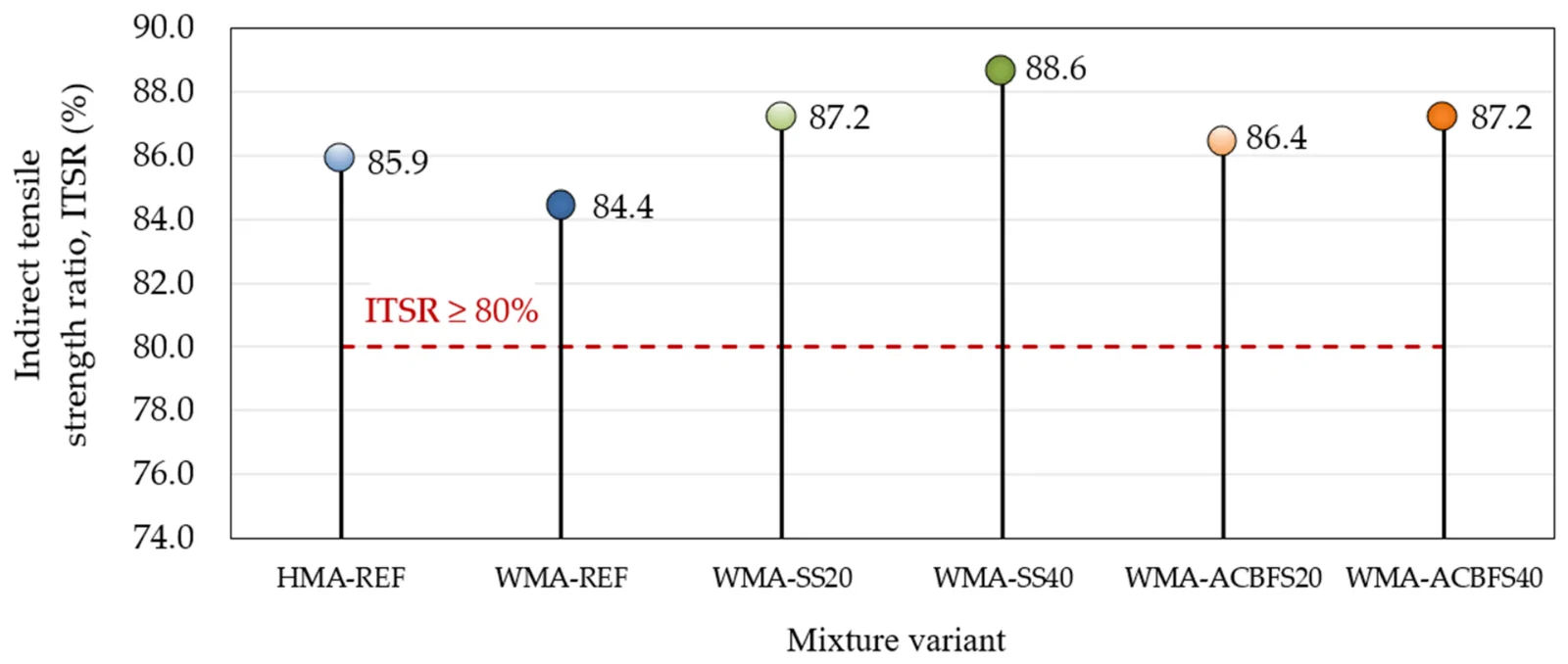
Indirect tensile strength ratio of the investigated mixtures. The color coding is the same as in Figure 6. The dashed horizontal line indicates the minimum required ITSR value.

Descriptive statistics for indirect tensile strength determined on dry specimens, ITS_d_, together with Tukey’s HSD multiple-comparison results, are presented in Table 8. Corresponding results for specimens after water and freeze–thaw conditioning, ITS_w_, are summarized in Table 9. The distributions of both parameters and the identified homogeneous groups are shown in Figure 7. The indirect tensile strength ratio (ITSR) values are presented in Figure 8. The minimum required ITSR value for the intended application was 80% [28].

WMA-REF had a mean ITS_d_ approximately 9% lower than HMA-REF. Incorporation of slag aggregates caused a further decrease in strength, and the effect became more pronounced as the replacement level increased from 20% to 40%. Relative to WMA-REF, the reduction in mean ITS_d_ ranged from approximately 4% for WMA-SS20 to approximately 19% for WMA-ACBFS40. At both replacement levels, mixtures containing ACBFS had lower mean values than the corresponding SS mixtures. A similar trend was observed after conditioning. The highest mean ITS_w_ was obtained for HMA-REF and the lowest for WMA-ACBFS40. Relative to WMA-REF, incorporation of 20% SS produced little change in mean conditioned strength, whereas increasing the slag aggregate replacement level to 40%, particularly for ACBFS, resulted in a larger reduction. Variability was greater for conditioned specimens than for dry specimens. The coefficient of variation ranged from 5.69% to 11.77% for ITS_d_ and from 7.86% to 14.71% for ${ITS}_{w}$. The greatest relative variability in both conditions was observed for WMA-ACBFS20.

For ITS_d_, the Shapiro–Wilk test showed no significant departures from normality in any mixture group (p=0.316–0.986), and the Brown–Forsythe test indicated no violation of the homogeneity-of-variance assumption (p=0.712). One-way ANOVA showed significant differences among mixture variants in ITS_d_, F(5,42)=13.78, p<0.001, $\omega^{2}=0.571$. Tukey’s HSD test showed that HMA-REF did not differ significantly from WMA-REF but had significantly higher ITS_d_ than all slag-containing mixtures. WMA-REF did not differ significantly from the mixtures containing 20% SS or ACBFS, whereas the differences relative to WMA-SS40 and WMA-ACBFS40 were significant. Among the slag-containing mixtures, a significant difference was found between WMA-SS20 and WMA-ACBFS40.

For ITS_w_, the Shapiro–Wilk test likewise showed no significant departures from normality (p=0.208–0.950), and the Brown–Forsythe test indicated no violation of the homogeneity-of-variance assumption (p=0.833). One-way ANOVA showed significant differences among mixture variants in ITS_w_, F(5,42)=7.31, p<0.001, $\omega^{2}=0.397$. After conditioning, HMA-REF had significantly higher ITS_w_ than WMA-SS40, WMA-ACBFS20, and WMA-ACBFS40, whereas the differences relative to WMA-REF and WMA-SS20 were not statistically significant. WMA-REF differed significantly only from WMA-ACBFS40. The arrangement of homogeneous groups from “A” to “C”, with overlapping intermediate groups, indicates a gradual decrease in ITS_w_ without clear separation of all intermediate variants.

ITSR values ranged from 84.4% to 88.6%, and all mixtures therefore satisfied the minimum requirement of 80% [28]. The lowest value was obtained for WMA-REF and the highest for WMA-SS40. Incorporation of slag aggregates did not reduce ITSR below the values obtained for the reference mixtures. Increasing SS content from 20% to 40% increased ITSR from 87.2% to 88.6%, whereas the corresponding increase in ACBFS content raised ITSR from 86.4% to 87.2%. The higher ITSR values of the slag-containing mixtures indicate a lower relative loss of strength after conditioning. However, this does not indicate an improvement in absolute strength, because both ITSd and ITSw decreased as slag aggregate content increased.

### 3.4. Dynamic Modulus

**Figure 9:**
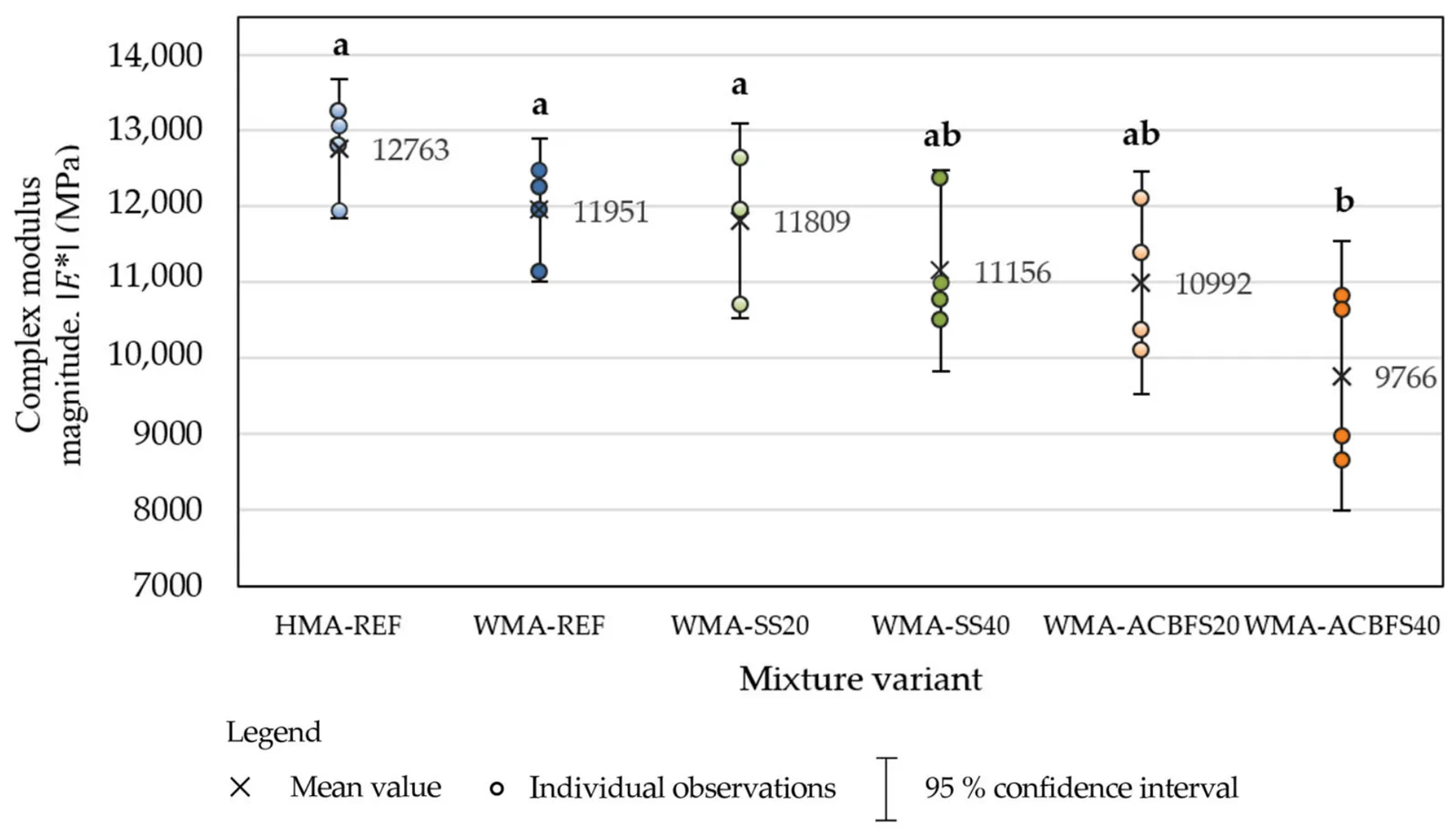
Dynamic modulus magnitude of the investigated mixtures. Lowercase letters denote homogeneous groups determined using Tukey’s HSD test at α=0.05. The color coding is the same as in Figure 6.

Descriptive statistics for dynamic modulus, |E*|, determined at 13 °C and 10 Hz, together with the results of Tukey’s HSD multiple comparisons, are presented in Table 10. Individual measurements, mean values, 95% confidence intervals for the means, and the identified homogeneous groups are shown in Figure 9.

WMA-REF had a mean |E*| 6.4% lower than HMA-REF. The difference between the two reference mixtures was not statistically significant. Incorporation of slag aggregates caused a further decrease in modulus, and this trend became more pronounced with increasing slag content and was more evident for ACBFS. Relative to WMA-REF, the reduction in mean |E*| ranged from 1.2% for WMA-SS20 to 18.3% for WMA-ACBFS40. The lowest mean |E*| was obtained for WMA-ACBFS40. Variability also increased as mean modulus decreased. The coefficient of variation increased from 4.51% for HMA-REF to 11.44% for WMA-ACBFS40.

The Shapiro–Wilk test showed no significant departures from normality in any mixture group (p=0.183–0.576), and the Brown–Forsythe test indicated no violation of the homogeneity-of-variance assumption (p=0.466). One-way ANOVA showed significant differences among mixture variants in |E*|, F(5,18)=6.09, p=0.002, $\omega^{2}=0.514$. Because of the small number of replicates in each group (n=4), the statistical results should be interpreted together with the individual observations and confidence intervals shown in Figure 9.

Tukey’s HSD test showed that WMA-ACBFS40 had significantly lower |E*| than HMA-REF, WMA-REF, and WMA-SS20. No significant differences were found between WMA-ACBFS40 and WMA-SS40 or WMA-ACBFS20.

### 3.5. Rutting Resistance

**Figure 10:**
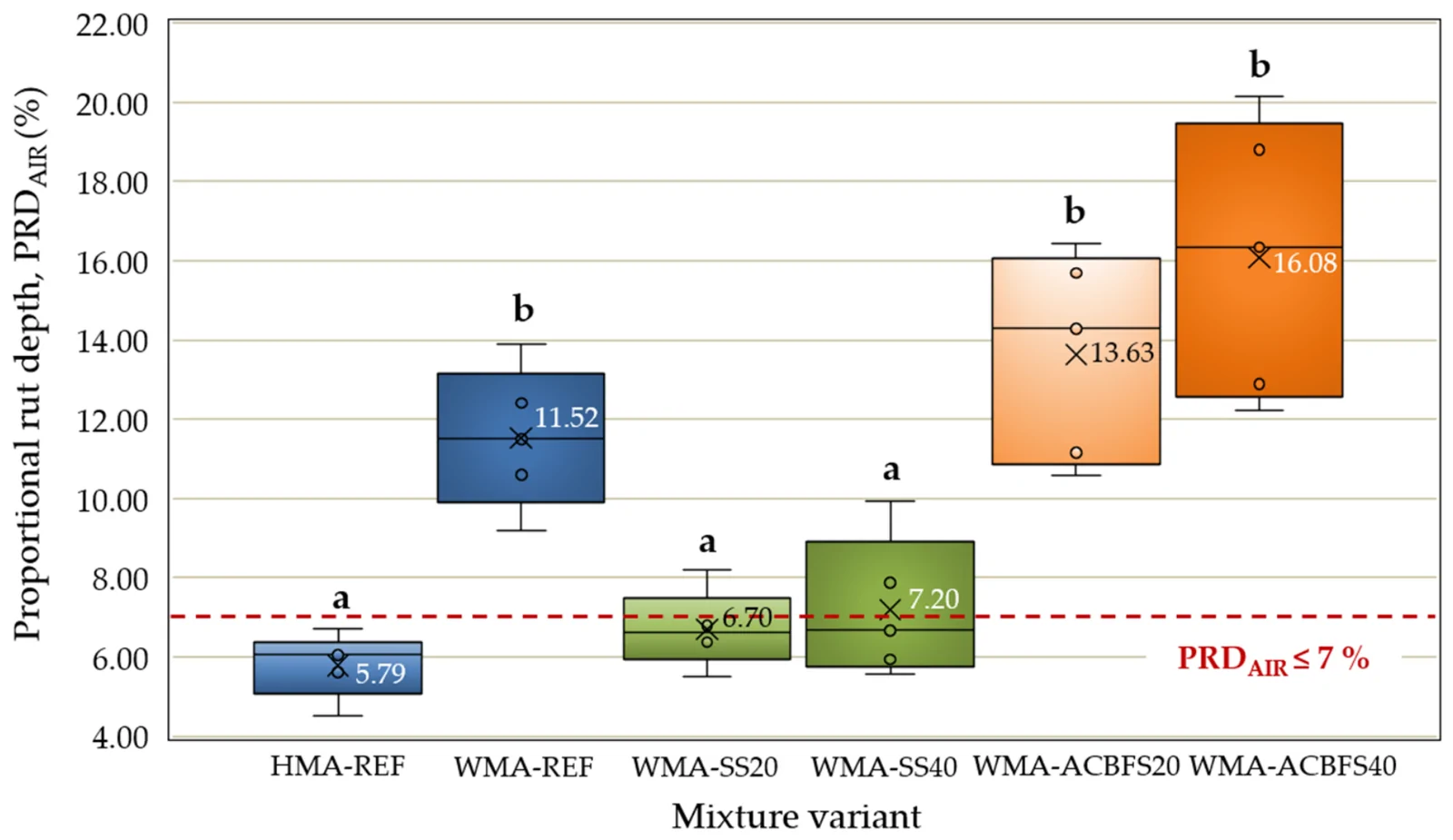
Proportional rut depth of the investigated mixtures. Lowercase letters denote homogeneous groups determined using the Games–Howell test at α=0.05. The boxplot elements and color coding follow those defined in Figure 6. The dashed horizontal line indicates the maximum specified PRD_AIR_ value.

**Figure 11:**
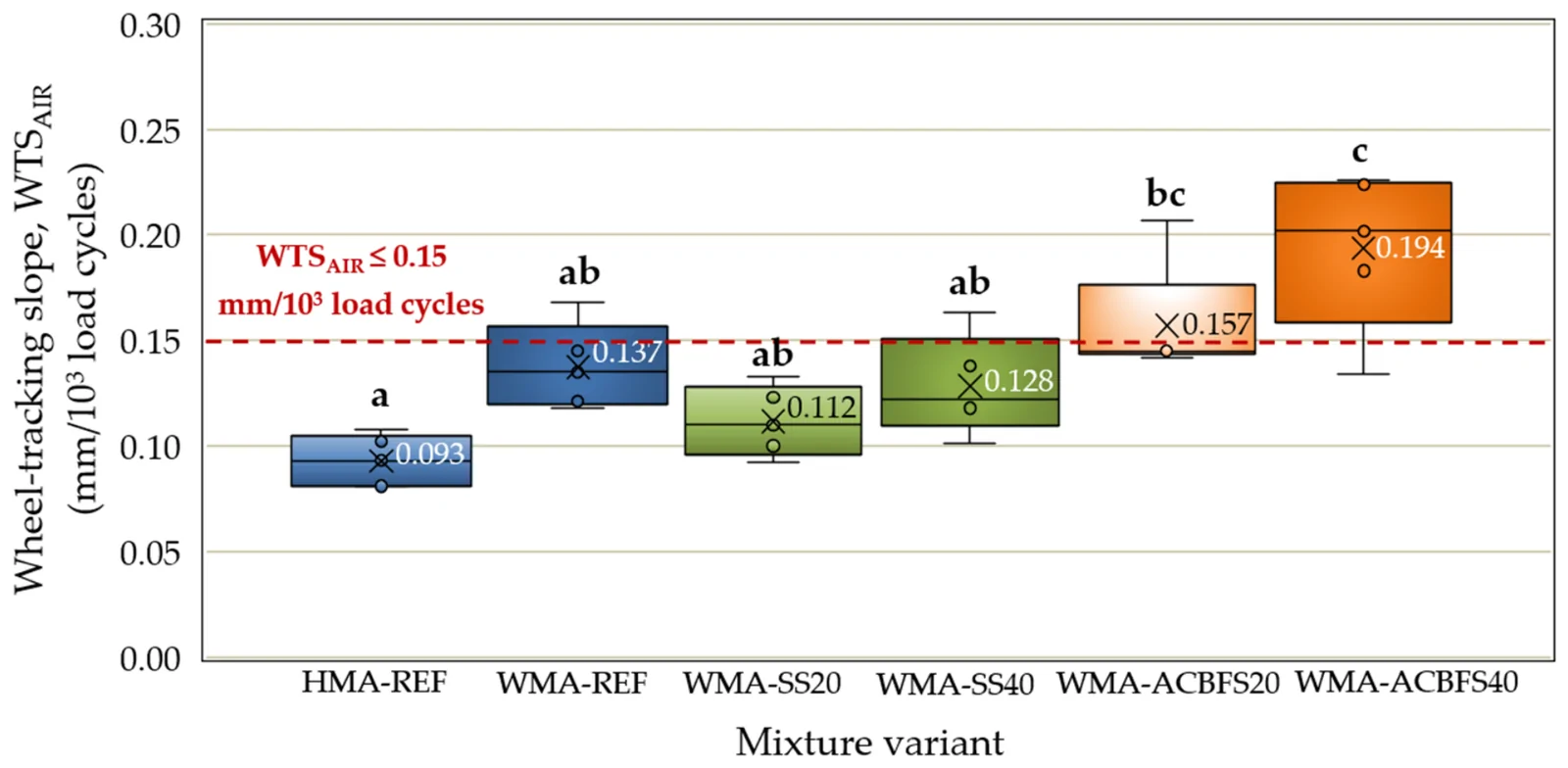
Wheel-tracking slope of the investigated mixtures. Lowercase letters denote homogeneous groups determined using Tukey’s HSD test at α=0.05. The boxplot elements and color coding follow those defined in Figure 6. The dashed horizontal line indicates the maximum specified WTS_AIR_ value.

Descriptive statistics for proportional rut depth, PRD_AIR_, and the results of Games–Howell pairwise comparisons are presented in Table 11. Result distributions, mean values, and homogeneous groups are shown in Figure 10. Corresponding results for wheel-tracking slope, WTS_AIR_, with Tukey’s HSD pairwise comparisons, are summarized in Table 12 and shown in Figure 11. According to the technical requirements [28], the maximum permitted values are 7.0% for PRD_AIR_ and 0.15 mm/10^3^ load cycles for WTS_AIR_.

WMA-REF had a mean PRD_AIR_ of 11.52%, nearly twice the 5.79% obtained for HMA-REF. Incorporation of SS reduced PRD_AIR_ relative to WMA-REF, whereas ACBFS produced the opposite trend, with the highest mean value observed for WMA-ACBFS40. HMA-REF and WMA-SS20 satisfied the PRD_AIR_ requirement of 7.0%, whereas WMA-SS40 slightly exceeded the limit, reaching 7.20%. WMA-REF and both ACBFS mixtures also failed to satisfy this criterion. The PRD_AIR_ results showed relatively high variability, particularly for WMA-SS40 and the ACBFS mixtures.

The Shapiro–Wilk test showed no significant departures from normality in any mixture group (p=0.351–1.000). The Brown–Forsythe test, however, indicated violation of the homogeneity-of-variance assumption (p=0.046). Welch’s analysis of variance was therefore applied and showed significant differences among mixture variants in PRD_AIR_, $F_{W}(5,10.83)=17.20$, p<0.001.

The Games–Howell test confirmed significantly lower PRD_AIR_ values for HMA-REF and the SS-containing mixtures than for WMA-REF and the ACBFS-containing mixtures. Despite the increase in mean value from 11.52% for WMA-REF to 16.08% for WMA-ACBFS40, the difference between these mixtures was not statistically significant, which may be associated with the greater variability of the WMA-ACBFS40 results.

The WTS_AIR_ results followed trends consistent with those observed for PRD_AIR_. The lowest mean value was obtained for HMA-REF, whereas WMA-REF was approximately 47% higher. Incorporation of SS reduced WTS_AIR_ relative to WMA-REF. Mean values were 0.112 mm/10^3^ load cycles for WMA-SS20 and 0.128 mm/10^3^ load cycles for WMA-SS40. The ACBFS-containing mixtures had the highest WTS_AIR_ values. WMA-ACBFS20 reached 0.157 mm/10^3^ load cycles, whereas WMA-ACBFS40 reached 0.194 mm/10^3^ load cycles. Mean values for HMA-REF, WMA-REF, and both SS-containing mixtures did not exceed the required 0.15 mm/10^3^ load cycles. The WTS_AIR_ results showed greater variability for the ACBFS mixtures, particularly WMA-ACBFS40.

The Shapiro–Wilk test showed no significant departures from normality in five of the six groups. A departure was found for WMA-ACBFS20 (p<0.001), associated with a single high WTS_AIR_ value. Given the balanced experimental design and homogeneity of variance confirmed by the Brown–Forsythe test (p=0.740), one-way ANOVA was applied. It showed significant differences among mixture variants in WTS_AIR_, F(5,24)=10.48, p<0.001, $\omega^{2}=0.613$. The consistency of the overall conclusion was confirmed by the Kruskal–Wallis test, H(5)=20.41, p=0.001.

Tukey’s HSD test showed that WMA-ACBFS20 had significantly higher WTS_AIR_ than HMA-REF but did not differ significantly from the remaining WMA mixtures. WMA-ACBFS40 differed significantly from HMA-REF, WMA-REF, WMA-SS20, and WMA-SS40, whereas the difference relative to WMA-ACBFS20 was not statistically significant.

Combined evaluation of both parameters indicates that SS improved the resistance of the WMA mixtures to permanent deformation relative to WMA-REF. Among the slag-containing mixtures, WMA-SS20 showed the most favorable results. In contrast, ACBFS, particularly at the 40% replacement level, increased both proportional rut depth and WTS_AIR_.

### 3.6. Effect of Slag Aggregate Type, Replacement Level, and Their Interaction on Mixture Properties

To provide an integrated assessment of the effects of the slag aggregates, a two-way analysis of variance was performed for the four WMA mixtures containing SS or ACBFS at replacement levels of 20% and 40%. The factors were slag aggregate type and virgin aggregate replacement level. A separate model including the main effects of both factors and their interaction was fitted for each property. HMA-REF and WMA-REF were excluded from the factorial analysis because, at zero slag content, the factor level defining slag type cannot be specified. ITSR was also excluded because it was calculated from mean ITS_d_ and ITS_w_ values rather than from independent individual observations. Model assumptions were assessed separately for this subset of data and were considered acceptable for the factorial analysis.

**Figure 12:**
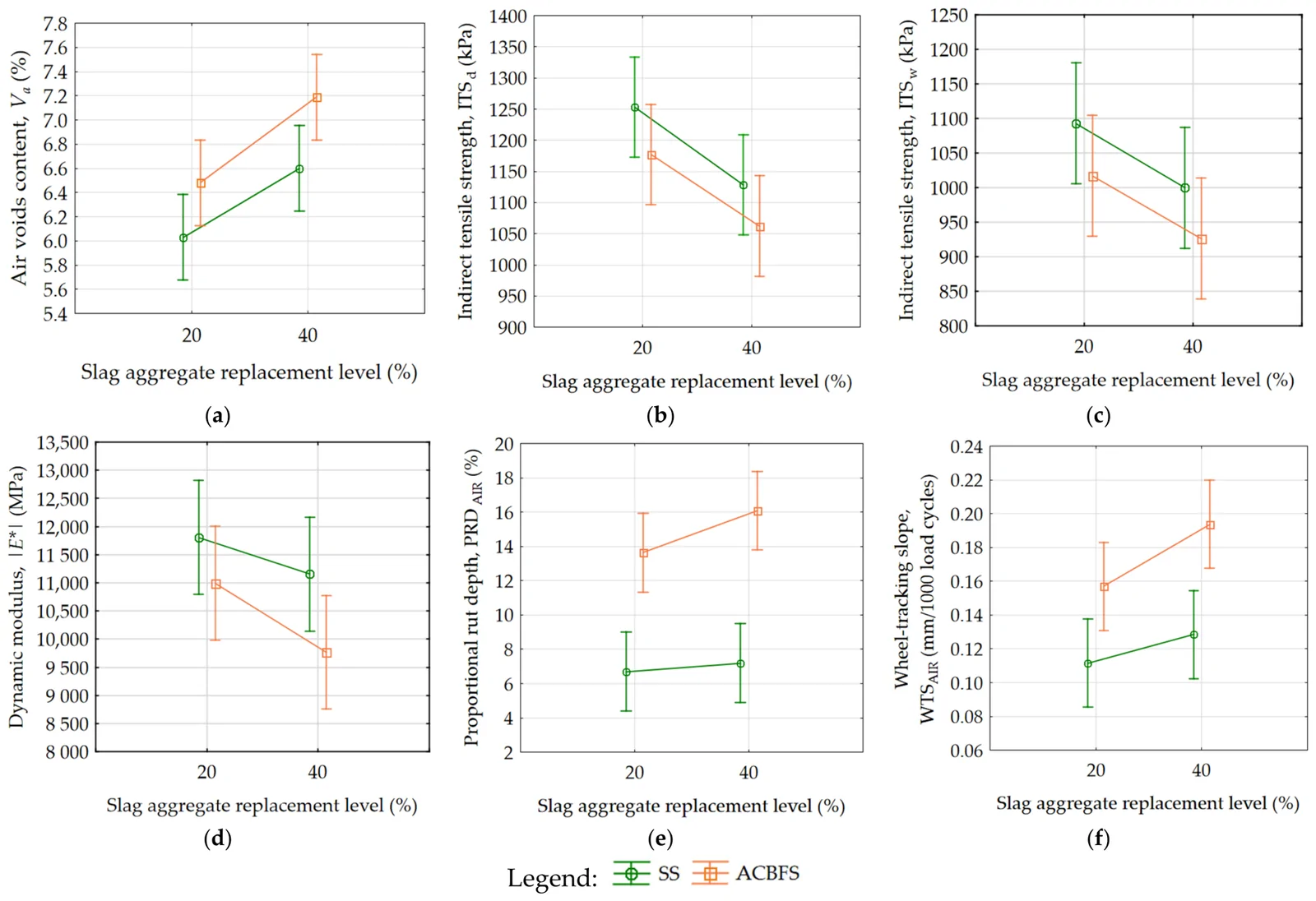
Estimated marginal means of the investigated properties as a function of slag aggregate type and replacement level: (**a**) air voids content, *V*_a_; (**b**) indirect tensile strength of dry specimens, ITS_d_; (**c**) indirect tensile strength of specimens after water and freeze–thaw conditioning, ITS_w_; (**d**) dynamic modulus, |E*|; (**e**) proportional rut depth, PRD_AIR_; and (**f**) wheel-tracking slope, WTS_AIR_. Vertical bars represent 95% confidence intervals.

**Figure 13:**
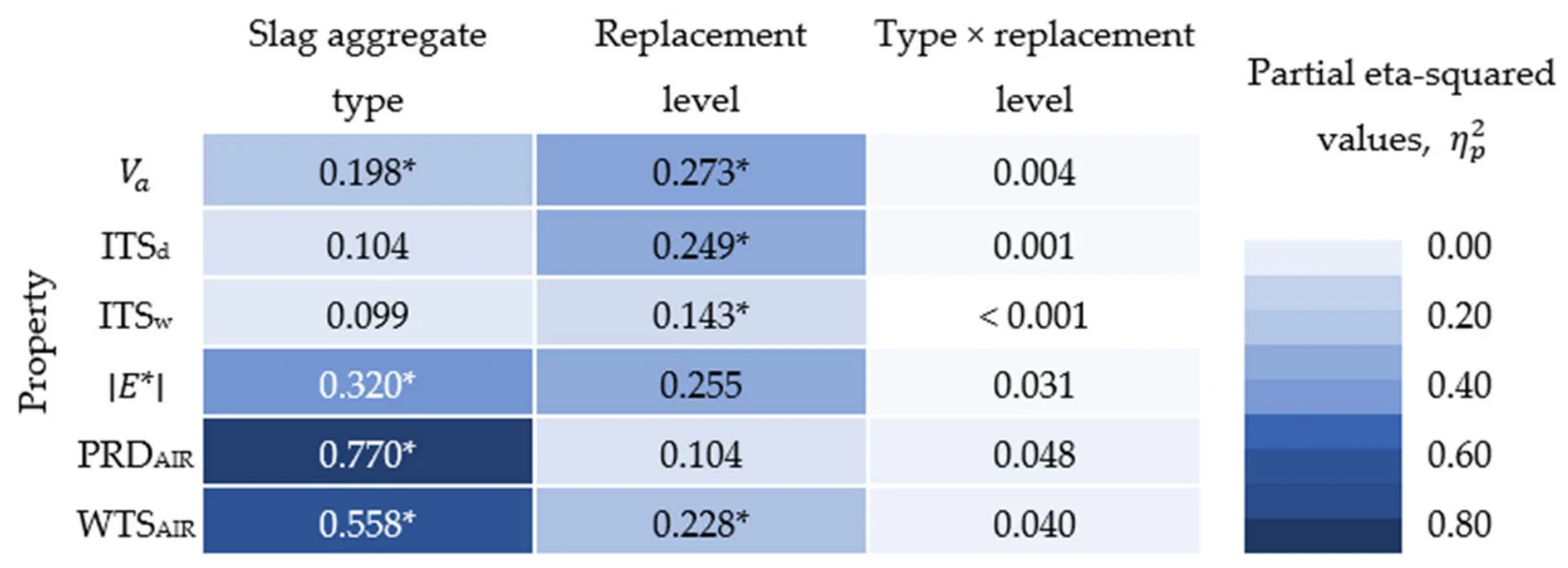
Heatmap of partial eta-squared values for the effects of slag aggregate type, replacement level, and their interaction on the investigated properties of slag-containing WMA mixtures. Asterisks denote statistically significant effects at α=0.05.

The results of the two-way analysis of variance are presented in Table 13. Estimated marginal means with 95% confidence intervals are shown in Figure 12. Effect sizes expressed as partial eta squared, $\eta_{p}^{2}$, are summarized in Figure 13. Darker heatmap cells correspond to larger $\eta_{p}^{2}$ values.

Slag aggregate type had a significant effect on *V_a_*, |E*|, PRD_AIR_ and WTS_AIR_. The largest effect sizes were obtained for the parameters describing resistance to permanent deformation. Mixtures containing ACBFS had higher values of both rutting parameters than mixtures containing SS. This indicates that, within the investigated range, resistance to permanent deformation depended more strongly on slag aggregate type than on increasing the replacement level from 20% to 40%.

A significant effect of aggregate type was also found for dynamic modulus. Mixtures containing SS had higher |E*| values than the ACBFS variants. Slag type also affected air voids content, with higher values obtained for the ACBFS mixtures. No significant effect of aggregate type was found for ITS_d_ or ITS_w_, although the mean values of both parameters were higher for mixtures containing SS.

Replacement level had a significant effect on *V_a_*, ITS_d_, ITS_w_ and WTS_AIR_. Increasing slag aggregate content from 20% to 40% increased air voids content and WTS_AIR_. At the same time, indirect tensile strength decreased both before conditioning and after water and freeze–thaw conditioning.

Increasing the replacement level was also accompanied by a decrease in $|E^{*}|$. This effect did not reach the adopted level of statistical significance despite a relatively large effect size. The result should be interpreted with caution because fewer dynamic modulus results were available in each group. For PRD_AIR_, increasing the replacement level from 20% to 40% increased the mean values, but the replacement-level effect was not statistically significant.

No significant interaction between slag aggregate type and replacement level was found for any investigated property (p=0.380–0.979). The corresponding effect sizes, expressed as partial eta squared ($\eta_{p}^{2}$), did not exceed 0.048. Thus, no statistical evidence was found that the effect of increasing the replacement level from 20% to 40% differed between SS and ACBFS. These effects should be interpreted within the adopted mixture-design framework because changes in slag type and replacement level were accompanied by adjustments in aggregate proportions and, for the SS mixtures, binder content resulting from the density correction.

Overall, slag aggregate type had the greatest influence on dynamic modulus and rutting resistance, whereas replacement level more strongly affected volumetric characteristics and indirect tensile strength. Within the investigated range, mixtures containing SS showed more favorable PRD_AIR_ and WTS_AIR_ values than the ACBFS variants.

### 3.7. Integrated Technical Assessment

An integrated technical assessment was performed against the technical requirements adopted for the intended use of the mixture in a pavement binder course and the assumed traffic loading level [28]. Mean values of the investigated parameters and the adopted limit values are summarized in Table 14. A mixture was considered to satisfy the full set of requirements included in the assessment only when all criteria listed in Table 14 were met.

The HMA-REF reference mixture satisfied all adopted requirements. For WMA-REF, air voids content, ITSR, and WTS_AIR_ were within the required ranges, whereas PRD_AIR_ was 11.52%, exceeding the maximum permitted value of 7.0%. WMA-REF therefore did not satisfy the full set of requirements included in the assessment.

Among the slag-containing mixtures, only WMA-SS20 satisfied all requirements included in the assessment. Its PRD_AIR_ and WTS_AIR_ values were 6.70% and 0.112 mm/10^3^ load cycles, respectively, while the requirements for *V_a_* and ITSR were also satisfied. The results confirm that replacing 20% of the virgin aggregate with SS reduced the susceptibility of WMA-REF to permanent deformation without loss of compliance with the remaining requirements included in the assessment. WMA-SS40 satisfied the requirements for *V_a_*, ITSR, and WTS_AIR_. However, its mean PRD_AIR_ was 7.20%, exceeding the limit by only 0.20 percentage points. WMA-SS40 therefore did not satisfy the full set of adopted criteria. The small exceedance of the limit also indicates the potential for further optimization of this variant at a 40% aggregate replacement level.

Both ACBFS-containing mixtures failed to satisfy the requirements for resistance to permanent deformation. For WMA-ACBFS20, PRD_AIR_ was 13.63%, while WTS_AIR_ was 0.157 mm/10^3^ load cycles. Both parameters increased further for WMA-ACBFS40. In addition, the air voids content of WMA-ACBFS40 was 7.19%, slightly exceeding the upper limit of the required range. Independently of these mixture-level results, the investigated ACBFS aggregate did not satisfy the LA30 requirement applicable to the target traffic loading and therefore was not eligible for the intended binder-course application. All investigated mixtures achieved ITSR values above the required 80%. The highest value was obtained for WMA-SS40 and the lowest for WMA-REF. Thus, resistance to water and freeze–thaw conditioning was not a limiting criterion for the suitability of any investigated variant.

Properties for which no direct limit values were specified in the adopted assessment system were used as supplementary comparative indicators. Mixtures containing SS had higher ITS_d_, ITS_w_, and |E*| values than the corresponding ACBFS variants. These results complement the compliance assessment based on parameters covered by the technical requirements.

The integrated assessment indicates that WMA-SS20 provided the most favorable balance of properties among the investigated slag-containing mixtures. Under the tested conditions, it was the only slag-containing variant that satisfied all mixture requirements included in the assessment.

## 4. Discussion

The results partially support the proposed hypothesis. Slag aggregate type and replacement level affected selected properties of the WMA mixtures, whereas no significant interaction between these factors was found for any investigated property. The two-way analysis showed significant effects of slag type on *V_a_*, |E*|, PRD_AIR_, and WTS_AIR_, while replacement level significantly affected *V_a_*, ITS_d_, ITS_w_, and WTS_AIR_. Thus, within the investigated range, there was no statistical evidence that increasing the replacement level from 20% to 40% affected the properties of SS- and ACBFS-containing mixtures differently. However, the effects of slag incorporation varied among the investigated properties. Several properties deteriorated as slag content increased, whereas SS improved rutting resistance relative to WMA-REF. Because the binder content was adjusted according to the aggregate blend density and the aggregate proportions were modified to maintain the required gradation, aggregate type, replacement level, binder content, and mixture composition varied simultaneously. Moreover, the mass-based definition of the replacement levels meant that equal nominal replacement levels did not correspond to equal aggregate volumes. Owing to the higher particle density of SS, the volumetric replacement levels were 16.4% at 20% mass replacement and 34.3% at 40% mass replacement, compared with 19.9% and 39.8% for ACBFS at the same nominal replacement levels. This difference may have contributed to the observed mixture response and should be considered when comparing the effects of SS and ACBFS. Therefore, the statistical effects should be interpreted as differences among the adopted mixture designs rather than as isolated causal effects of slag type or replacement level.

The comparison between HMA-REF and WMA-REF also highlights the importance of the baseline properties of the WMA mixture. The adopted WMA technology was accompanied by an increase in *V_a_* and decreases in ITS_d_, ITS_w_, and |E*|, with the largest differences observed for PRD_AIR_ and WTS_AIR_. These changes cannot, however, be attributed solely to reduced temperature or bitumen foaming because the reference mixtures also differed in adhesion promoter content. The comparison should therefore be interpreted as the combined effect of the adopted WMA technology. Previous studies indicate that the properties of foamed-bitumen WMA may change because of aging and further densification during service [9,10], whereas long-term behavior may be comparable to that of HMA when service conditions are considered [8]. The laboratory findings of the present study therefore require subsequent field verification.

Differences between the SS- and ACBFS-containing mixtures can be partly related to the properties of the aggregates used. ACBFS had lower density, higher water absorption, and lower resistance to fragmentation than SS, while SEM observations showed a more developed fine-scale surface texture with local voids and depressions. These characteristics are consistent with the higher *V_a_*, lower |E*|, and lower rutting resistance of the ACBFS-containing mixtures, but they do not allow the observed changes to be attributed to a single mechanism. Differences in air voids content may also have contributed to the observed differences in dynamic modulus and rutting resistance. However, these parameters were determined on separate specimen sets using different compaction procedures. Therefore, the contribution of air voids content could not be isolated statistically using the present dataset. Bitumen absorption by the aggregate was not measured in this study, and the potential effect of the greater ACBFS porosity on binder availability in the mixture therefore requires direct verification. Moura et al. [52] demonstrated the importance of slag aggregate surface characteristics for binder adhesion and mixture moisture resistance, whereas Lei et al. [53] highlighted the important role of the steel slag–bitumen interfacial zone. Mixture response may therefore result from the combined effects of aggregate mechanical properties, surface structure, and binder–aggregate interaction conditions.

All investigated mixtures satisfied the ITSR requirement, and the slag-containing variants exhibited a lower relative loss of strength after conditioning than the reference mixtures. This result should, however, be interpreted together with ITS_d_ and ITS_w_, both of which decreased as slag content increased. Favorable moisture resistance of SS-containing WMA has also been reported in previous studies [17,19], although the WMA technologies used in those studies differed from the approach adopted here.

The most favorable effect of SS was observed for resistance to permanent deformation. Incorporation of 20% SS reduced PRD_AIR_ and WTS_AIR_ relative to WMA-REF, and WMA-SS20 satisfied all technical requirements included in the assessment. This finding is consistent with Liu et al. [12], who demonstrated that SS angularity and content affect contact characteristics within the aggregate skeleton, with the effect also depending on mixture gradation. Dondi et al. [11] and Hassan et al. [13] also reported favorable resistance to permanent deformation in asphalt mixtures containing SS. Comparable improvements in the mechanical performance of SS-containing WMA were reported by Ameri et al. [16], Masoudi et al. [18], Amelian et al. [19], and Ziaee and Behnia [20]. Zhu et al. [54] showed that the performance of asphalt mixtures containing steel slag depends on the replacement level and slag surface condition, confirming the need to optimize slag content for a given mixture. Increasing SS content to 40% did not produce further improvement. The PRD_AIR_ value for WMA-SS40 was 7.20%, exceeding the adopted limit by only 0.20 percentage points. Such a small exceedance indicates a need for further optimization of this variant rather than exclusion of the possibility of using 40% SS.

Long-term use of SS also requires consideration of volume stability. Residual free CaO and MgO may undergo hydration and cause delayed expansion [5]. Aging, weathering, or soaking before use can reduce this risk by promoting hydration of these constituents. In the present study, the SS had been stockpiled for several months before testing and showed a volume expansion of 0.5% according to EN 1744-1 [40], satisfying the adopted volume-stability requirement. Nevertheless, long-term field verification remains appropriate.

Less favorable results were obtained for the ACBFS-containing mixtures. Increasing its content was accompanied by a decrease in |E*|, while PRD_AIR_ and WTS_AIR_ were higher than in the corresponding SS variants. These results should not be interpreted as evidence that ACBFS is generally unsuitable for WMA. Ruíz-Ibarra et al. [23] reported improved resistance to permanent deformation for WMA containing 12.5% blast furnace slag as a replacement of the coarse aggregate fraction, whereas less favorable performance was observed at a higher replacement level of 24%. Their mixtures were also produced using a chemical WMA additive. These contrasting results indicate that mixture performance depends on the replacement level, the properties of the specific slag aggregate, and the WMA technology used. The importance of the dosage basis is also supported by Rondón-Quintana et al. [22], who showed that mass- and volume-based replacement with blast furnace slag can lead to different mixture behavior. Further optimization of ACBFS variants should therefore consider the basis of dosage, gradation, and binder content. Kunaev et al. [55] also investigated selective crushing as a method for improving the physical and mechanical properties of blast furnace slag aggregate for asphalt concrete, indicating that the material can be optimized before the mixture design stage.

The results show that incorporating industrially derived aggregate does not necessarily impair WMA performance. With appropriate selection of slag type and replacement level, the required properties can be maintained and, for some parameters, improved relative to the reference mixture. Potential environmental benefits may result from the use of industrial by-products as aggregate substitutes and from the reduced production temperatures of WMA. However, these benefits cannot be quantified from the present study alone. Transport distances and the higher particle density of SS may increase material transport requirements and should be considered in an environmental assessment. The need to consider technical performance together with environmental aspects has also been emphasized by Díaz-Piloneta et al. [56], who evaluated steel slag as a road aggregate from both technical and environmental perspectives. A complete assessment of environmental benefits, however, requires a separate life-cycle analysis accounting for the specific materials, production technology, and expected service life [6,15].

The 20% and 40% replacement levels adopted in this study should not be regarded as universal limits for SS and ACBFS use. This investigation covered one asphalt concrete type, one paving-grade bitumen, one source of each slag aggregate, and one baseline WMA composition. In addition, dynamic modulus was determined at a single temperature and loading frequency, and the results therefore provide a comparative assessment of mixture stiffness under the adopted test conditions rather than a full characterization of temperature- and frequency-dependent behavior. Future studies should extend the dynamic modulus testing to a broader range of temperatures and loading frequencies. Of particular importance, WMA-REF itself did not satisfy the adopted PRD_AIR_ requirement. Further studies should therefore determine whether an optimized baseline WMA mixture with greater resistance to permanent deformation would allow a higher SS content and broader use of ACBFS. Optimization should address gradation, binder content and binder absorption by the aggregate, compaction conditions, and foaming parameters. Future studies should determine air voids content for the same specimens used in dynamic modulus and rutting tests and consider comparisons or normalization at comparable air void levels. The investigated solutions should also be evaluated in other pavement layers and under lower traffic loading. Further studies should include X-ray computed tomography of compacted specimens to assess air void structure and particle contacts, together with X-ray diffraction of the aggregates to identify mineral phases. The experimental program should also address fatigue and low-temperature behavior and include field verification under actual production, compaction, and service conditions.

## 5. Conclusions

Based on the laboratory investigation and statistical analyses, the following conclusions were drawn:

- Slag aggregate type significantly affected selected properties of asphalt concrete intended for pavement binder courses and produced with foamed bitumen. Steel slag aggregate provided more favorable properties than air-cooled blast furnace slag aggregate, particularly in terms of stiffness and resistance to permanent deformation.
- Among the slag-containing WMA mixtures, the most favorable properties were obtained when 20% of the virgin aggregate was replaced with steel slag aggregate. This mixture satisfied all technical requirements included in the assessment and exhibited greater resistance to permanent deformation than the reference mixture produced with foamed bitumen.
- Increasing the steel slag aggregate replacement level to 40% did not provide further improvement, although the exceedance of the proportional rut depth requirement was small. This result supports further optimization of this variant rather than its exclusion.
- Mixtures containing air-cooled blast furnace slag aggregate had higher air voids content, lower stiffness, and lower resistance to permanent deformation. At the same time, all investigated mixtures satisfied the requirement for resistance to water and freeze–thaw conditioning.
- Partial replacement of virgin aggregate with slag aggregates can be technically justified for selected slag types and replacement levels. Under the investigated conditions, the mixture containing 20% SS was the only slag-containing variant that satisfied the full set of adopted mixture requirements.
- From a practical perspective, 20% SS replacement appears to be a suitable solution for foamed-bitumen WMA used in pavement binder courses. Higher SS contents and ACBFS may also be considered for applications with lower traffic loading, provided that the mixtures are appropriately optimized and satisfy the relevant technical requirements.
- This study was limited to one asphalt concrete type, one paving-grade bitumen, one source of each slag aggregate, and one baseline WMA composition. Therefore, the findings provide a basis for further application and optimization of slag-containing WMA, but their transfer to other materials, mixture designs, and traffic conditions requires additional verification.

## Figures and Tables

**Table 2:** Selected properties of slag aggregates considered in accordance with the adopted technical requirements [26].

Property	Test Method	Unit	Slag Aggregate Type	
			SS	ACBFS
Particle size distribution	EN 933-1 [30]	—	Figure 3b	Figure 3b
Flakiness index, FI	EN 933-3 [34]	%	8	6
Shape index, SI	EN 933-4 [35]	%	10	7
Fines content	EN 933-1 [30]	%	6.1	3.9
Methylene blue value, MBF	EN 933-9 [36]	g/kg	0.9	1.1
Los Angeles coefficient, LA	EN 1097-2 [37]	%	23	35
Micro-Deval coefficient, MDE	EN 1097-1 [38]	%	17	22
Apparent particle density, *ρ_a_*	EN 1097-6 [31]	Mg/m^3^	3.44	2.71
Oven-dried particle density, *ρ_rd_*	EN 1097-6 [31]	Mg/m^3^	3.18	2.54
Saturated surface-dry particle density, *ρ_ssd_*	EN 1097-6 [31]	Mg/m^3^	3.31	2.61
Water absorption after 24 h, WA_24_	EN 1097-6 [31]	%	1.6	2.6
Mass loss after the freeze–thaw test	EN 1367-1 [39]	%	0.3	1.3
Volume stability	EN 1744-1 [40]	%	0.5	n.d.
Dicalcium silicate disintegration	EN 1744-1 [40]	—	n.d.	No disintegration
Iron disintegration	EN 1744-1 [40]	—	n.d.	No disintegration
Coarse, lightweight contaminants	EN 1744-1 [40]	%	0.0	0.0

Note: n.d.—not determined; — no unit applicable. According to the adopted technical requirements [26], dicalcium silicate disintegration and iron disintegration are applicable to ACBFS, whereas volume stability is applicable to SS.

**Table 3:** Basic properties of the 35/50 asphalt binder with different adhesion promoter contents.

Property	Unit	Test Method	Asphalt Binder		
			35/50	35/50 + 0.3% Adhesion Promoter	35/50 + 0.6% Adhesion Promoter
Penetration at 25 °C	0.1 mm	EN 1426 [42]	37.1 ± 1.21	41.9 ± 1.62	43.5 ± 0.95
Softening point	°C	EN 1427 [43]	53.1 ± 0.35	52.5 ± 0.14	52.7 ± 0.06
Fraass breaking point	°C	EN 12593 [44]	−13.9 ± 0.61	−13.6 ± 1.55	−12.8 ± 0.61
Penetration index	—	EN 12591 [41]	−1.1	−1.0	−0.9
Plasticity range	°C	—	67.0	66.1	65.5

Note: — not applicable. The adhesion promoter content is expressed as a percentage of asphalt binder mass. Values are presented as mean ± standard deviation.

**Table 4:** Composition of the investigated aggregate blends and asphalt mixtures.

Mixture Component	Mixture Variant											
	HMA-REF		WMA-REF		WMA-SS20		WMA-SS40		WMA-ACBFS20		WMA-ACBFS40	
	Mass (%)	Vol. (%)	Mass (%)	Vol. (%)	Mass (%)	Vol. (%)	Mass (%)	Vol. (%)	Mass (%)	Vol. (%)	Mass (%)	Vol. (%)
Mineral filler, % of aggregate blend	4.0	4.0	4	4.0	4.0	4.2	3.0	3.3	5.0	5.0	6.0	6.0
0/2 mm virgin aggregate, %	16.0	15.8	16	15.8	13.0	13.5	17.0	18.4	11.0	10.9	0.0	0.0
0/4 mm virgin aggregate, %	16.0	16.0	16	16.0	13.0	13.6	2.0	2.2	7.0	7.0	9.0	9.0
2/5 mm virgin aggregate, %	13.0	13.2	13	13.2	9.0	9.5	7.0	7.8	13.0	13.2	7.0	7.1
5/8 mm virgin aggregate, %	13.0	13.2	13	13.2	9.0	9.5	7.0	7.8	13.0	13.2	9.0	9.1
8/16 mm virgin aggregate, %	38.0	37.8	38	37.8	32.0	33.3	24.0	26.2	31.0	30.8	29.0	29.0
Slag aggregate SS 0/16 mm, %	0.0	0.0	0.0	0.0	20.0	16.4	40.0	34.3	0.0	0.0	0.0	0.0
Slag aggregate ACBFS 0/16 mm, %	0.0	0.0	0.0	0.0	0.0	0.0	0.0	0.0	20.0	19.9	40.0	39.8
Total aggregate blend, %	100	100	100	100	100	100	100	100	100	100	100	100
Asphalt binder content, % of asphalt mixture	4.5	—	4.5	—	4.3	—	4.1	—	4.5	—	4.5	—
Adhesion promoter content, % of asphalt binder mass	0.3	—	0.6	—	0.6	—	0.6	—	0.6	—	0.6	—
FWC, % of asphalt binder mass	—	—	3.0	—	3.0	—	3.0	—	3.0	—	3.0	—

Note: — not applicable.

**Table 5:** Test methods, specimen numbers, and experimental conditions used for the investigated asphalt mixtures.

Property	Test Method	Specimen Type	Number per Mixture	Main Test Conditions	Result	Unit
Air voids content	EN 12697-5 [45], EN 12697-6 [46], and EN 12697-8 [47]	Loose mixture and Marshall specimens	10 compacted specimens	75 blows per side; storage for 72 h at room temperature	*V_a_*	%
Indirect tensile strength and water and freeze–thaw resistance	EN 12697-12 [48], EN 12697-23 [49] and technical requirements [28]	Marshall specimens	8 dry + 8 conditioned	Water conditioning and one freeze–thaw cycle	ITS_d_	kPa
					ITS_w_	kPa
					ITSR	%
Dynamic modulus	AASHTO T 342-11 [50]	Gyratory-compacted specimens, cored and trimmed to Ø100 × 150 mm	4	Air voids content: target ±0.5%; 13 °C; 10 Hz; axial strain ≤ 75 µε	|E*|	MPa
Rutting resistance	EN 12697-22 [51]	Laboratory roller-compacted slabs, 300 × 400 × 60 mm	5	6 h conditioning at 60 °C; Method B in air; small device; 10,000 load cycles	PRD_AIR_	%
					WTS_AIR_	mm/10^3^ load cycles

**Table 6:** Local semi-quantitative elemental composition of the investigated slag aggregate surfaces determined by EDS analysis.

Slag Type	Sample	Element Composition (wt.%)													Total
		C	O	Na	Mg	Al	Si	S	K	Ca	Ti	Cr	Mn	Fe	
SS	1	3.03	31.14	0.15	2.63	2.34	8.19	0.40	-	19.27	0.14	0.34	4.62	27.75	100
	2	1.87	22.71	0.06	1.20	6.20	5.86	0.27	-	22.05	0.44	0.42	4.67	34.25	100
ACBFS	1	2.98	35.49	0.12	2.63	2.55	16.43	3.51	0.85	35.44	-	-	-	-	100
	2	3.81	39.03	0.17	1.84	2.11	16.14	2.49	0.57	33.84	-	-	-	-	100

Note: - not detected.

**Table 7:** Descriptive statistics and Tukey’s HSD-adjusted p-values for pairwise comparisons of the air voids content of the investigated mixtures.

Mixture Variant	Air Voids Content, *V_a_* (%)			*p*					
	Mean	SD	CV (%)	{1}	{2}	{3}	{4}	{5}	{6}
HMA-REF {1}	4.54	0.20	4.43		0.032 *	<0.001 *	<0.001 *	<0.001 *	<0.001 *
WMA-REF {2}	5.20	0.28	5.36	0.032 *		0.003 *	<0.001 *	<0.001 *	<0.001 *
WMA-SS20 {3}	6.03	0.44	7.38	<0.001 *	0.003 *		0.091	0.285	<0.001 *
WMA-SS40 {4}	6.60	0.50	7.63	<0.001 *	<0.001 *	0.091		0.993	0.073
WMA-ACBFS20 {5}	6.48	0.52	8.06	<0.001 *	<0.001 *	0.285	0.993		0.017 *
WMA-ACBFS40 {6}	7.19	0.70	9.73	<0.001 *	<0.001 *	<0.001 *	0.073	0.017 *	

Legend: 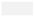
—shaded cells—diagonal cells; *p*—*p*-value; *—p≤0.05, statistically significant difference between the compared mean values; values without an asterisk—p>0.05, no statistically significant difference between the compared mean values; SD—standard deviation; CV—coefficient of variation.

**Table 8:** Descriptive statistics and Tukey’s HSD-adjusted p-values for pairwise comparisons of indirect tensile strength determined on dry specimens of the investigated mixtures.

Mixture Variant	Indirect Tensile Strength, ITS_d_ (kPa)			*p*					
	Mean	SD	CV (%)	{1}	{2}	{3}	{4}	{5}	{6}
HMA-REF {1}	1441.50	82.09	5.69		0.142	0.010 *	<0.001 *	<0.001 *	<0.001 *
WMA-REF {2}	1310.63	92.27	7.04	0.142		0.875	0.013 *	0.128	<0.001 *
WMA-SS20 {3}	1253.04	95.16	7.59	0.010 *	0.875		0.179	0.691	0.008 *
WMA-SS40 {4}	1128.25	90.50	8.02	<0.001 *	0.013 *	0.179		0.933	0.800
WMA-ACBFS20 {5}	1177.13	138.56	11.77	<0.001 *	0.128	0.691	0.933		0.255
WMA-ACBFS40 {6}	1062.26	114.63	10.79	<0.001 *	<0.001 *	0.008 *	0.800	0.255	

Legend: 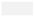
—shaded cells—diagonal cells; *p*—*p*-value; *—p≤0.05, statistically significant difference between the compared mean values; values without an asterisk—p>0.05, no statistically significant difference between the compared mean values; SD—standard deviation; CV—coefficient of variation.

**Table 9:** Descriptive statistics and Tukey’s HSD-adjusted *p*-values for pairwise comparisons of indirect tensile strength determined on conditioned specimens of the investigated mixtures.

Mixture Variant	Indirect Tensile Strength, ITS_w_ (kPa)			*p*					
	Mean	SD	CV (%)	{1}	{2}	{3}	{4}	{5}	{6}
HMA-REF {1}	1238.35	97.35	7.86		0.201	0.126	0.002 *	0.004 *	<0.001 *
WMA-REF {2}	1106.04	94.72	8.56	0.201		0.999	0.427	0.620	0.031 *
WMA-SS20 {3}	1092.90	108.44	9.92	0.126	0.999		0.572	0.760	0.054
WMA-SS40 {4}	999.73	110.46	11.05	0.002 *	0.427	0.572		0.999	0.783
WMA-ACBFS20 {5}	1017.12	149.65	14.71	0.004 *	0.620	0.760	0.999		0.598
WMA-ACBFS40 {6}	926.25	108.50	11.71	<0.001 *	0.031 *	0.054	0.783	0.598	

Legend: 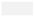
—shaded cells—diagonal cells; *p*—*p*-value; *—p≤0.05, statistically significant difference between the compared mean values; values without an asterisk—p>0.05, no statistically significant difference between the compared mean values; SD—standard deviation; CV—coefficient of variation.

**Table 10:** Descriptive statistics and Tukey’s HSD-adjusted p-values for pairwise comparisons of the dynamic modulus of the investigated mixtures.

Mixture Variant	Dynamic Modulus, ∣E*∣ (MPa)			*p*					
	Mean	SD	CV (%)	{1}	{2}	{3}	{4}	{5}	{6}
HMA-REF {1}	12,763	576.01	4.51		0.735	0.592	0.115	0.068	<0.001 *
WMA-REF {2}	11,951	587.45	4.92	0.735		0.999	0.750	0.586	0.016 *
WMA-SS20 {3}	11,809	808.27	6.84	0.592	0.999		0.869	0.730	0.027 *
WMA-SS40 {4}	11,156	830.03	7.44	0.115	0.750	0.869		0.999	0.218
WMA-ACBFS20 {5}	10,992	923.50	8.40	0.068	0.586	0.730	0.999		0.334
WMA-ACBFS40 {6}	9766	1117.05	11.44	<0.001 *	0.016 *	0.027 *	0.218	0.334	

Legend: 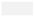
—shaded cells—diagonal cells; *p*—*p*-value; *—p≤0.05, statistically significant difference between the compared mean values; values without an asterisk—p>0.05, no statistically significant difference between the compared mean values; SD—standard deviation; CV—coefficient of variation.

**Table 11:** Descriptive statistics and Games–Howell-adjusted p-values for pairwise comparisons of the proportional rut depth among the investigated mixtures.

Mixture Variant	Proportional Rut Depth, PRD_AIR_ (%)			*p*					
	Mean	SD	CV (%)	{1}	{2}	{3}	{4}	{5}	{6}
HMA-REF {1}	5.79	0.81	13.99		0.006 *	0.620	0.618	0.011 *	0.013 *
WMA-REF {2}	11.52	1.79	15.53	0.006 *		0.013 *	0.038 *	0.687	0.233
WMA-SS20 {3}	6.70	0.98	14.63	0.620	0.013 *		0.991	0.017 *	0.018 *
WMA-SS40 {4}	7.20	1.76	24.46	0.618	0.038 *	0.991		0.021 *	0.018 *
WMA-ACBFS20 {5}	13.63	2.65	19.44	0.011 *	0.687	0.017 *	0.021 *		0.804
WMA-ACBFS40 {6}	16.08	3.50	21.77	0.013 *	0.233	0.018 *	0.018 *	0.804	

Legend: 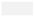
—shaded cells—diagonal cells; *p*—*p*-value; *—p≤0.05, statistically significant difference between the compared mean values; values without an asterisk—p>0.05, no statistically significant difference between the compared mean values; SD—standard deviation; CV—coefficient of variation.

**Table 12:** Descriptive statistics and Tukey’s HSD-adjusted p-values for pairwise comparisons of wheel-tracking slope of the investigated mixtures.

Mixture Variant	Wheel-Tracking Slope, WTS_AIR_ (mm/10^3^ Load Cycles)			*p*					
	Mean	SD	CV (%)	{1}	{2}	{3}	{4}	{5}	{6}
HMA-REF {1}	0.093	0.01	10.75		0.080	0.832	0.238	0.005 *	<0.001 *
WMA-REF {2}	0.137	0.02	14.56	0.080		0.565	0.991	0.800	0.015 *
WMA-SS20 {3}	0.112	0.02	17.92	0.832	0.565		0.883	0.070	<0.001 *
WMA-SS40 {4}	0.128	0.02	15.58	0.238	0.991	0.883		0.456	0.004 *
WMA-ACBFS20 {5}	0.157	0.03	19.11	0.005 *	0.800	0.070	0.456		0.204
WMA-ACBFS40 {6}	0.194	0.04	20.64	<0.001 *	0.015 *	<0.001 *	0.004 *	0.204	

Legend: 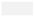
—shaded cells—diagonal cells; *p*—*p*-value; *—p≤0.05, statistically significant difference between the compared mean values; values without an asterisk—p>0.05, no statistically significant difference between the compared mean values; SD—standard deviation; CV—coefficient of variation.

**Table 13:** Results of the two-way analysis of variance for the effects of slag aggregate type, replacement level, and their interaction on the properties of slag-containing WMA mixtures.

Property	Slag Aggregate Type		Replacement Level		Type × Replacement Level	
	*F*	*p*	*F*	*p*	*F*	*p*
*V_a_*	8.909	0.005 *	13.496	<0.001 *	0.161	0.690
ITS_d_	3.248	0.082	9.266	0.005 *	0.016	0.901
ITS_w_	3.066	0.091	4.662	0.040 *	0.001	0.979
∣E*∣	5.658	0.035 *	4.099	0.066	0.381	0.549
PRD_AIR_	53.595	<0.001 *	1.854	0.192	0.814	0.380
WTS_AIR_	20.223	<0.001 *	4.733	0.045 *	0.659	0.429

Legend: *F*—test statistic; *p*—*p*-value; *—p≤0.05, statistically significant effect; values without an asterisk—p>0.05, no statistically significant effect. The numerator degree of freedom was 1 for all effects. The denominator degrees of freedom were 36 for *V_a_*, 28 for ITS_d_ and ITS_w_, 12 for |E*|, and 16 for PRD_AIR_ and WTS_AIR_.

**Table 14:** Integrated assessment of the investigated mixtures against the technical requirements [28] adopted for the intended application.

Mixture Type	*V_a_* (%)	ITSR (%)	PRD_AIR_ (%)	WTS_AIR_ (mm/10^3^ Load Cycles)	Overall Compliance
Requirement	4.0–7.0	≥80	≤7.0	≤0.15	All requirements met
HMA-REF	4.54 ✓	85.9 ✓	5.79 ✓	0.093 ✓	✓
WMA-REF	5.20 ✓	84.4 ✓	11.52 ✗	0.137 ✓	✗
WMA-SS20	6.03 ✓	87.2 ✓	6.70 ✓	0.112 ✓	✓
WMA-SS40	6.60 ✓	88.6 ✓	7.20 ✗	0.128 ✓	✗
WMA-ACBFS20	6.48 ✓	86.4 ✓	13.63 ✗	0.157 ✗	✗
WMA-ACBFS40	7.19 ✗	87.2 ✓	16.08 ✗	0.194 ✗	✗

Legend: ✓—requirement met; ✗—requirement not met. Overall compliance indicates that all technical requirements included in the assessment were satisfied.

## Data Availability

The original contributions presented in this study are included in the article. Further inquiries can be directed to the corresponding author.

## Abbreviations

The following abbreviations are used in this manuscript:

ACBFS	Air-cooled blast furnace slag
ANOVA	Analysis of variance
CV	Coefficient of variation
EDS	Energy-dispersive X-ray spectroscopy
ER	Expansion ratio
ESAL_100 kN_	Equivalent single-axle load based on a 100 kN standard axle
FWC	Foaming water content
HL	Half-life
HMA	Hot-mix asphalt
HSD	Honestly significant difference
ITS_d_	Indirect tensile strength of dry specimens
ITS_w_	Indirect tensile strength of specimens after water and freeze–thaw conditioning
ITSR	Indirect tensile strength ratio
PRD_AIR_	Proportional rut depth determined in the wheel-tracking test in air
SD	Standard deviation
SEM	Scanning electron microscopy
SS	Steel slag
WMA	Warm-mix asphalt
WTS_AIR_	Wheel-tracking slope determined in air
ZAF	Atomic number, absorption, and fluorescence correction
